# An Overview of Variational Autoencoders for Source Separation, Finance, and Bio-Signal Applications

**DOI:** 10.3390/e24010055

**Published:** 2021-12-28

**Authors:** Aman Singh, Tokunbo Ogunfunmi

**Affiliations:** Department of Electrical & Computer Engineering, Santa Clara University, Santa Clara, CA 95053, USA; asingh9@scu.edu

**Keywords:** variational autoencoders, deep learning, volatility surfaces, speech source separation, EEG, EMG, ECG, generative models

## Abstract

Autoencoders are a self-supervised learning system where, during training, the output is an approximation of the input. Typically, autoencoders have three parts: Encoder (which produces a compressed latent space representation of the input data), the Latent Space (which retains the knowledge in the input data with reduced dimensionality but preserves maximum information) and the Decoder (which reconstructs the input data from the compressed latent space). Autoencoders have found wide applications in dimensionality reduction, object detection, image classification, and image denoising applications. Variational Autoencoders (VAEs) can be regarded as enhanced Autoencoders where a Bayesian approach is used to learn the probability distribution of the input data. VAEs have found wide applications in generating data for speech, images, and text. In this paper, we present a general comprehensive overview of variational autoencoders. We discuss problems with the VAEs and present several variants of the VAEs that attempt to provide solutions to the problems. We present applications of variational autoencoders for finance (a new and emerging field of application), speech/audio source separation, and biosignal applications. Experimental results are presented for an example of speech source separation to illustrate the powerful application of variants of VAE: VAE, β-VAE, and ITL-AE. We conclude the paper with a summary, and we identify possible areas of research in improving performance of VAEs in particular and deep generative models in general, of which VAEs and generative adversarial networks (GANs) are examples.

## 1. Introduction

One of the distinct traits of human intelligence is the ability to imagine and synthesize. Generative modeling in machine learning aims to train algorithms to synthesize completely new data, such as audio, text, and images; it does so by estimating the density of the data, and then sampling from that estimated density. The deep learning [1] revolution has led to breakthroughs in generative modeling with deep generative models such as variational autoencoders, generative stochastic networks, neural autoregressive models, and generative adversarial networks.

Variational autoencoders combine Bayesian variational inference with deep learning [2]; like the autoencoder, it has an encoder and decoder, but it aims to learn the probability distribution through amortized variational inference and the reparameterization trick. Information theory is a key component of variational inference because it involves minimizing the KL Divergence between the posterior distribution and variational posterior. The generative adversarial network is a framework for training two models (a discriminator and a generator) simultaneously with an adversarial process.

There has been lots of research on deep generative models for image, text, and audio generation. Generative adversarial networks (GANs) tend to outperform variational autoencoders in image fidelity, while a variational autoencoder is more stable and is better for estimating the probability distribution itself. Neural autoregressive models are powerful at density estimation but often slower than VAEs in sampling. Often times, these different frameworks have been integrated to compliment the different strengths and ameliorate the weaknesses, such as with the adversarial autoencoder, VAE-GAN, VAE with inverse autoregressive flow, and PixelVAE.

Source separation, especially blind source separation, has long been a problem of interest in the signal processing community. The cocktail party problem is a common example of the blind source separation problem. It is when a listener is at a cocktail party where there are many people speaking concurrently, and the listener must try to follow one of the conversations. It seems like an easy task for humans, but, for computers, it is more difficult. Source separation has applications in music/speech/audio data, EEG signals, ECG signals, and image processing. In the past, methods like Independent Component Analysis (ICA) and Non Negative Matrix Factorization (NNMF) have been the state-of-the-art methods for this problem. More recently, deep learning methods have improved our solution, including variational autoencoders, generative adversarial networks, and recurrent neural networks.

For applications in finance (which is relatively new), VAEs are used to generate synthetic volatility surfaces for options trading.

Bio-signal applications of VAE include detection of serious diseases using electrocardiogram (ECG) signals, data augmentation of bio-signals and improving electroencephalography (EEG)-based speech recognition systems, etc.

The key contributions of our paper include:

(1) A general overview of autoencoders and VAEs (2) A comprehensive survey of applications of the variational autoencoders for speech source separation, data augmentation and dimensionality reduction in finance, and biosignal analysis. (3) A comprehensive survey of variational autoencoder variants. (4) Experiments and analysis of results in speech source separation using various VAE models. (5) While multiple survey papers have covered the VAE [3,4], this paper has a special focus on time series/signal processing applications and information-theoretic interpretations.

The paper is organized as follows: Section 2 provides a general background information and Section 3 discusses Variational Inference. Section 4 presents the Variational Autoencoder while Section 5 discusses Problems with the VAE. Several variants of the VAEs are presented in Section 6. Three interesting applications of VAEs are discussed in Section 7. Experimental results on speech source separation are discussed in Section 8 and Section 9 concludes the paper.

### Notations

The following notation will be used throughout this paper:

- Lower case *p*, *q*, *f*, γ, ψ, or p(.), q(.), f(.), γ(.), ψ(.), to denote probability density functions (PDFs) or probability mass functions (PMFs).
- Random variables are written in lower case italicized letters, for example, *x*, with the exception of ϵ, which will represent both a random variable and its instance.
- Deterministic scalar terms, including realizations of random variables, are written in lower case letters, for example, x. Greek alphabets α, β, θ, and ϕ will also denote deterministic scalar terms. Deterministic scalar terms that are not realizations of random variables can also be written in upper case letters, such as N.
- Random vectors are written in lower case italicized bold letters, for example, x.
- if we have a random vector x, then its jth component will be noted $x_{j}$.
- Ordinary vectors are written in lowercase bold letters or bold Greek alphabets θ, μ, and ϕ. A realization of a random vector x will be written as x.
- Matrices are written in uppercase bold italics, such as W. I denotes the identity matrix.
- If we have two random variables *x* and *y* with probability functions p(x) and p(y), we can write their joint probability function as p(x,y). Their conditional probability distribution function is written as p(x|y).
- If we have a PMF/PDF p(x=x), p(x) is the shorthand notation. For p(x|y=y), p(x|y) is the shorthand. For p(x=x|y=y), p(x|y) is the shorthand.
- If we have p(.;θ) or $p_{\theta }\left(x\right)$, this denotes that the PDF/PMF *p* is parameterized by θ—similarly with $q_{\phi }\left(z\right)$. However, there are exceptions in some contexts. $p_{x}\left(x\right)$ will be shorthand for a distribution p(x) associated with random variable *x*; $p_{z}\left(z\right)$ will be shorthand for a distribution *p* associated with random variable *z*; and $p_{x|z}\left(x|z_{i}\right)$ denotes a conditional distribution of random variable *x* given *z*=$z_{i}$. The term $p_{\left(x,y\right)}\left(x,y\right)$ is a joint PDF/PMF between random variables *x* and *y*. The term $p_{\left(x,z\right)}\left(x,z\right)$ is a joint PDF/PMF between random variables *x* and *z*. In Section 2.8, $p_{g}\left(z\right)$ would represent the distribution that is the input to the generator in the GAN, while $p_{d}\left(x\right)$ represents the data distribution.
- range(*x*) denotes the range of random variable *x* and dom(*x*) denotes the domain of random variable *x*.
- If we have a dataset $X={\left\{x^{\left(i\right)}\right\}}_{i=1}^{N}$, where there are N independent and identically distributed (i.i.d.) realizations/observations of some continuous or discrete random variable *x*, the ith observation is denoted as $x^{\left(i\right)}$.
- If we have a dataset $X={\left\{x^{\left(i\right)}\right\}}_{i=1}^{N}$, where there are N i.i.d. realizations/observations of some continuous or discrete random vector x, the ith observation is denoted as $x^{\left(i\right)}$. If this our dataset, then $x_{j}^{\left(i\right)}$ represents the jth component of the ith observation.
- diag(x) is a diagonal matrix, with the diagonal values being the values of vector x and det (W) is the determinant of matrix W.

## 2. Background

In this section, we summarize the prerequisite information for understanding variational autoencoders and its many extensions.

### 2.1. Distances and Information-Theoretic Measures

Information theory is key to machine learning, from the usage of information-theoretic measures for the loss functions [5,6,7] to use for analysis through the information bottleneck framework [8,9,10]. It is also key to the development of the VAE [2]. Therefore, it is recommended to know key measures in information theory.

#### 2.1.1. Shannon’s Entropy

If you have a random variable *x*, Shannon’s Entropy is a measure of uncertainty of *x*, with a PDF of p(x)[11]. It can also be thought of as a way to measure the uncertainty in a random vector or random process x, which has a joint PDF p(x). It is also a generalization of the variance of a process.

Discrete Shannon’s Entropy, for discrete random variable *x*, with a PMF p(x), is defined as:

(1) $$H\left(x\right)=-\sum_{x\in range(x)}p\left(x\right)\log\left(p\left(x\right)\right)$$

Continuous (Differential) Shannon’s Entropy, with the PDF p(x), for random variable *x*, is given by

(2) $$H\left(x\right)=-\int_{range(x)}p\left(x\right)\log\left(p\left(x\right)\right)dx$$

We can also rewrite both discrete and differential Entropy as:

(3) $$H\left(x\right)=-E_{p(x)}\left[\log\left(p\left(x\right)\right)\right]$$

#### 2.1.2. Shannon’s Joint Entropy

The Joint Entropy H(x,y) of a pair of discrete random variables (x,y) with a joint distribution $p_{\left(x,y\right)}\left(x,y\right)$ is defined as

(4) $$H\left(x,y\right)=-\sum_{x\in range(x)}\sum_{y\in range(y)}p_{\left(x,y\right)}\left(x,y\right)\log\left(p_{\left(x,y\right)}\left(x,y\right)\right)$$

We can also write the Joint Entropy as

(5) $$H\left(x,y\right)=-E_{p(x,y)}\left[\log\left(p\left(x,y\right)\right)\right]$$

and

(6) H(x,y)=H(y)+H(x|y)

If we have $x=\left(x_{1},x_{2},\ldots ,x_{n}\right)∼p\left(x_{1},x_{2},\ldots ,x_{n}\right)=p\left(x\right)$, where x is a discrete random vector, then

(7) $$H\left(x_{1},x_{2},\ldots ,x_{n}\right)=-\sum_{x_{1},x_{2},\ldots ,x_{n}}p\left(x_{1},x_{2},\ldots ,x_{n}\right)\log\left(p\left(x_{1},x_{2},\ldots ,x_{n}\right)\right)$$

(8) $$H\left(x\right)=-\sum_{x\in range(x)}p\left(x\right)\log\left(p\left(x\right)\right)$$

If we have $x=\left(x_{1},x_{2},\ldots ,x_{n}\right)∼p\left(x_{1},x_{2},\ldots ,x_{n}\right)=p\left(x\right)$, where x is a continuous random vector, then

(9) $$H\left(x_{1},\ldots ,x_{n}\right)=-\int p\left(x_{1},\ldots ,x_{n}\right)\log\left(p\left(x_{1},\ldots ,x_{n}\right)\right)dx_{1}\ldots dx_{n}$$

(10) $$H\left(x\right)=-\int p\left(x\right)\log\left(p\left(x\right)\right)dx$$

#### 2.1.3. Shannon’s Conditional Entropy

If we have $\left(x,y\right)∼p_{\left(x,y\right)}\left(x,y\right)$, where *x* and *y* are discrete random variables, the conditional Entropy H(y|x) is:

$$\begin{array}{cc} H(y|x) & =\sum_{x\in range(x)}p\left(x\right)H\left(y|x=x\right)\\ & =-\sum_{x\in range(x)}p\left(x\right)\sum_{y\in range(y)}p_{y|x}\left(y|x\right)\log\left(p_{y|x}\left(y|x\right)\right)\\ & =-\sum_{x\in range(x)}\sum_{y\in range(y)}p_{\left(x,y\right)}\left(x,y\right)\log\left(p_{y|x}\left(y|x\right)\right)\\ & =-E_{p(x,y)}\left[\log\left(p\left(y|x\right)\right)\right] \end{array}$$

The conditional Entropy H(x|y) is:

$$\begin{array}{cc} H(x|y) & =\sum_{y\in range(y)}p\left(y\right)H\left(x|y=y\right)\\ & =-\sum_{y\in range(y)}p\left(y\right)\sum_{x\in range(x)}p_{x|y}\left(x|y\right)\log\left(p_{x|y}\left(x|y\right)\right)\\ & =-\sum_{y\in range(y)}\sum_{x\in range(x)}p_{\left(x,y\right)}\left(x,y\right)\log\left(p_{x|y}\left(x|y\right)\right)\\ & =-E_{p(x,y)}\left[\log\left(p\left(x|y\right)\right)\right] \end{array}$$

Conditional Entropy can be thought of as the “*expected value of the entropies of the conditional distributions, averaged over the conditioning random variable*” [11].

If (x,y) are continuous random variables with the joint PDF p(x,y), the conditional differential Entropy H(x|y) is

H(x|y)=−∫p(x,y)log(p(x|y))dxdy.

Since the joint PDF has the property p(x|y)=p(x,y)/p(y), H(x|y)=H(x,y)−H(y).

#### 2.1.4. Kullback–Leiber (KL) Divergence

If you have two probability distributions, *p* and *q*, the KL Divergence measures the similarity between the two distributions; however, it is asymmetric [11]. It is also non-negative.

For discrete random variables with PMFs *p* and *q*, the discrete KL Divergence is given by

(11) $$D_{KL}\left(p∥q\right)=\sum_{x\in range(x)}p\left(x\right)\log\left(\frac{p(x)}{q(x)}\right)$$

If *p* and *q* are distributions of a continuous random variable *x*, the continuous KL Divergence is given by

(12) $$D_{KL}\left(p∥q\right)=\int_{-\infty }^{\infty }p\left(x\right)\log\left(\frac{p(x)}{q(x)}\right)dx$$

We can also write the KL Divergence as

(13) $$D_{KL}\left(p∥q\right)=E_{p(x)}\left[\log\left(\frac{p(x)}{q(x)}\right)\right]$$

(14) $$D_{KL}\left(q∥p\right)=E_{q(x)}\left[\log\left(\frac{q(x)}{p(x)}\right)\right]$$

KL Divergence can be used when we are approximating a probability distribution *p* with another probability distribution *q*. We can use $D_{KL}\left(p∥q\right)$, called the forward KL, or $D_{KL}\left(q∥p\right)$, which is called the reverse KL. Minimizing the forward KL with respect to the approximate distribution *q* is called moment projection [12]. In the case where *p* is positive but *q* is $0,\log\left(\frac{p}{q}\right)$ becomes *∞*, so then the support of *p* is overestimated in the approximation *q*. Minimizing the reverse KL with respect to the approximate distribution *q* is called information projection. In the case where *q* is positive but *p* is 0, $\log\left(\frac{q}{p}\right)$ becomes *∞*, so the approximation *q* does not include any input where *p* is 0.

#### 2.1.5. Mutual Information

Mutual Information of random variables *x* and *y*, denoted I(x;y), measures the information that *x* and *y* share and the dependence between them [11]. Intuitively, it is how much knowing one random variable decreases uncertainty in the other one; this can be seen by the following formula:

(15) I(x;y)=H(y)−H(y|x)=H(x)−H(x|y)

Mutual Information can also be written as

(16) I(x;y)=H(x)+H(y)−H(x,y)

If *x* and *y* are discrete random variables, their discrete Mutual Information is given by

(17) $$I\left(x;y\right)=\sum_{y\in range(y)}\sum_{x\in range(x)}p_{\left(x,y\right)}\left(x,y\right)\log\left(\frac{p_{\left(x,y\right)}\left(x,y\right)}{p_{x}\left(x\right)p_{y}\left(y\right)}\right)$$

(18) $$I\left(x;y\right)=\sum_{y\in range(y)}\sum_{x\in range(x)}p_{\left(x,y\right)}\left(x,y\right)\log\left(\frac{p_{\left(x,y\right)}\left(x,y\right)}{p_{x}\left(x\right)p_{y}\left(y\right)}\right)$$

where $p_{\left(x,y\right)}\left(x,y\right)$ is the joint PMF between *x* and *y*. If *x* and *y* are continuous random variables, their continuous Mutual Information is

(19) $$I\left(x;y\right)={\;\int \int \;}_{range(x),range(y)}p_{\left(x,y\right)}\left(x,y\right)\log\left(\frac{p_{\left(x,y\right)}\left(x,y\right)}{p_{x}\left(x\right)p_{y}\left(y\right)}\right)dxdy$$

I(x;y)=0 if and only if *x* and *y* are independent. Mutual Information is non-negative and symmetric.

#### 2.1.6. Cross-Entropy

For two PMFs p and q, the Cross-Entropy is defined as:

(20) $$H\left(p,q\right)=-\sum_{x\in range(x)}p\left(x\right)\log\left(q\left(x\right)\right)$$

#### 2.1.7. Jensen–Shannon (JS) Divergence

Given PDFs *p* and *q*, we have $\psi =\frac{1}{2}\left(p+q\right)$. The JS Divergence is

(21) $$\mathrm{JSD}\left(p∥q\right)=\frac{1}{2}D_{KL}\left(p∥\psi \right)+\frac{1}{2}D_{KL}\left(q∥\psi \right)$$

This is also a symmetric measure.

#### 2.1.8. Renyi’s Entropy

Renyi’s Entropy is a generalization of Shannon’s Entropy [6]. Discrete Renyi’s Entropy for PMF p(x) is given by

(22) $$h_{\alpha }\left(X\right)=\frac{1}{\alpha -1}\log\left(\sum_{k=1}^{N}\left(p^{\alpha }\left(x\right)\right)\right),\alpha >0$$

Continuous Renyi’s Entropy for PDF p(x) is given by

(23) $$h_{\alpha }\left(x\right)=\frac{1}{\alpha -1}\log\left(\int \left(p^{\alpha }\left(x\right)dx\right)\right),\alpha >0$$

When α→1, Renyi’s Entropy converges to Shannon’s Entropy. When α=2, it becomes quadratic entropy. The term $V_{\alpha }\left(x\right)$ is called information potential:

(24) $$V_{\alpha }\left(x\right)=\sum_{k=1}^{N}p^{\alpha }\left(x\right)=\sum_{i=1}^{N}p^{\alpha -1}\left(x\right)p\left(x\right)=E\left[p^{\alpha -1}\left(x\right)\right]$$

If α = 2, it becomes the quadratic information potential (QIP):

(25) $$V_{\alpha }\left(x\right)=\sum_{k=1}^{N}p^{\alpha }\left(x\right)=\sum_{i=1}^{N}p^{\alpha -1}\left(x\right)p\left(x\right)=E\left[p^{\alpha -1}\left(x\right)\right]$$

We first look at the continuous Quadratic Entropy, which is a case of α-Renyi Entropy where α= 2:

(26) $$h_{2}\left(x\right)=-\log\left(\int p^{2}\left(x\right)dx\right)=-\log\left(E\left[p\left(x\right)\right]\right)$$

$V_{2}\left(x\right)=E_{p(x)}\left[p\left(x\right)\right]$ is the QIP; it is the expected value of the PDF if *x* is continuous, or PMF if *x* is discrete. From this point on, all information potentials will be QIPs. In addition, the subscripts will denote the PDF/PMF associated with the QIP; $V_{p}$ will be the QIP associated with *p*.

#### 2.1.9. Renyi’s Cross-Entropy

Renyi’s Cross-Entropy for two PDFs is given by:

(27) $$h_{2}\left(p,q\right)=-\log\left(\int p(x)q(x)dx\right)$$

The cross information potential is given by:

(28) $$V_{2}\left(p,q\right)=\left(\int p(x)q(x)dx\right)$$

From this point, all information potentials in this paper will be quadratic. In addition, the subscripts will denote the PDF/PMF associated with the QIP; $V_{p}$ will be the QIP associated with *p*; $V_{q}$ will be the QIP associated with *q*; $V_{c}$ will be the cross information potential associated with *p* and *q*.

#### 2.1.10. Renyi’s α-Divergence

For two PMFs, p(x) and q(x), the formula is:

(29) $$\begin{array}{ccc} D_{\alpha }\left(p\left(x\right);q\left(x\right)\right)=\frac{1}{\alpha -1}\log\left(\sum_{x\in range(x)}\sum_{y\in range(y)}p\left(x\right){\left(\frac{p(x)}{q(x)}\right)}^{\alpha -1}\right) & & \\ =\frac{1}{\alpha -1}\log\left(\sum_{x\in range(x)}\sum_{y\in range(y)}\frac{p^{\alpha }\left(x\right)}{q^{\alpha -1}\left(x\right)}\right) \end{array}$$

When α→1, it converges to KL Divergence.

For two PDFs p(x) and q(x), the formula is:

(30) $$\begin{array}{ccc} D_{\alpha }\left(p\left(x\right);q\left(x\right)\right)=\frac{1}{\alpha -1}\log\left(\int p\left(x\right){\left(\frac{p(x)}{q(x)}\right)}^{\alpha -1}dx\right) & & \\ =\frac{1}{\alpha -1}\log\left(\int \frac{{\left(p\left(x\right)\right)}^{\alpha }}{{\left(q\left(x\right)\right)}^{\alpha -1}}dx\right) \end{array}$$

#### 2.1.11. Euclidean Divergence

The Euclidean Divergence between PDFs p(x) and q(x) is given by the following formula:

(31) $$\begin{array}{ccc} D_{ED}\left(p\left(x\right);q\left(x\right)\right)=\int {\left(p\left(x\right)-q\left(x\right)\right)}^{2}dx & & \\ =\int {\left(p\left(x\right)\right)}^{2}dx+\int {\left(q\left(x\right)\right)}^{2}dx-2\int \left(p\left(x\right)q\left(x\right)\right)dx \end{array}$$

If we want to express the Euclidean Divergence between *p* and *q* in terms of QIP, it is given by:

(32) $$D_{ED}\left(p(x)∥q(x)\right)=V_{p}+V_{q}-2V_{c}$$

The Euclidean Divergence is a symmetric measure.

#### 2.1.12. Cauchy–Schwarz Divergence

The Cauchy–Schwarz (CS) Divergence, for probability density functions p(x) and q(x), is given by the following formula [6]:

(33) $$D_{CS}\left(p\left(x\right);q\left(x\right)\right)=\frac{-1}{2}\log\left(\frac{{\left(\int p(x)q(x)dx\right)}^{2}}{\int p^{2}\left(x\right)dx\int q^{2}\left(x\right)dx}\right)$$

If we have PDFs p(x) and q(x), and want to express the CS Divergence between them in terms of QIP, it is given by:

(34) $$D_{CS}\left(p(x)∥q(x)\right)=\log\left(\frac{V_{p}V_{q}}{V_{c}^{2}}\right)$$

Unlike KL Divergence and Renyi’s α-Divergence, CS Divergence is a symmetric measure.

### 2.2. Monte Carlo

Monte Carlo methods [13,14] are methods using random simulations; they are often used to estimate integrals. This is useful in statistics for estimating expected values. In machine learning, it is especially useful for the case of gradient estimation.

Given the fact that we have an integrable function $g:{\left[0,1\right]}^{d}\mapsto R$, we can look at the following integral [14]:

$$A=\int_{{\left[0,1\right]}^{d}}g\left(x\right)dx$$

If the domain of the integral is in $R^{d}$, the change of variables can change the domain to ${\left[0,1\right]}^{d}$.

We can generate an i.i.d sequence $\left\{u_{1},\ldots ,u_{N}\right\}$ from a standard uniform distribution over ${\left[0,1\right]}^{d}$. Then, the Monte Carlo estimator is given by

(35) $$A_{N}=\frac{1}{N}\sum_{n=1}^{N}g\left(u_{n}\right)$$

We can also use a more general Monte Carlo estimator for integration [15]. We can find a PDF *f* of random variable $z\in {\left[0,1\right]}^{d}$ such that *f* > 0 on ${\left[0,1\right]}^{d}$ and $\int_{{\left[0,1\right]}^{d}}f\left(x\right)dx=1$.

Given that h(x)=g(x)/f(x), our integral becomes

$$A=\int_{{\left[0,1\right]}^{d}}h\left(x\right)f\left(x\right)dx=E\left[h\left(z\right)\right]=E_{f}\left[h\left(z\right)\right]$$

The Monte Carlo estimator is then given by

(36) $$A_{N}=\frac{1}{N}\sum_{k=1}^{N}h\left(x_{k}\right)$$

Thus, the steps are

(1) sample i.i.d sequence $\{x_{1},x_{2}\ldots x_{N}\}∼f$
(2) Plug i.i.d. sequence into the estimator given by Equation (36).

If we set f(x) =1 over the region ${\left[0,1\right]}^{d}$, we arrive at Equation (35). From the Law of Large Numbers, $A_{N}$ for both Monte Carlo estimators converge to A when n →∞, and the convergence rate does not depend on dimension d; this provides an advantage over traditional integration.

### 2.3. Autoencoders

The Autoencoder is a self supervised learning algorithm that is used for lossy data compression [16]. The compression is specific to the data that is used to train the model.

**Figure 1:**
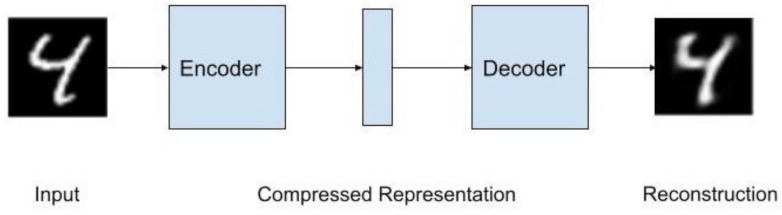
Autoencoder with MNIST input. The input is fed into the encoder. The encoder outputs a compressed representation, which is then inputted into the decoder. The decoder outputs a reconstruction of the input.

There is an encoder that creates a compressed representation; this representation goes into the decoder and outputs a reconstructed input. This algorithm test label is the data input itself. Autoencoders can be considered a nonlinear Principal Component Analysis (PCA). Often times, the compressed representation has a smaller dimension than the input and output. Figure 1 shows the architecture of an Autoencoder with the MNIST data set as the data.

The encoder and decoder are typically multilayered perceptrons (MLPs), but they can be replaced with convolutional neural networks, which becomes a Convolutional Autoencoder. The convolutional autoencoder is better with reconstructing image data. The use of convolution in deep learning actually refers to what is known as cross-correlation in signal processing terminology [1]. The LSTM-Autoencoder uses LSTMs instead of MLPs for the encoder and decoder.

One important variation of the Autoencoder is the Denoising Autoencoder (DAE) [17]; the DAE is used to clean data that is corrupted by noise. Random noise is added to the input, but the reconstruction loss is between the clean input and the output. Some noise that can be added includes salt & pepper noise, additive white Gaussian noise (AWGN), and masking noise.

Discrete-time white noise is a zero mean discrete-time random process with finite variance whose samples are serially uncorrelated random variables. AWGN is discrete-time white noise that is Gaussian and additive. Additive implies it is added to the original signal. We add AWGN to the original signal/image x. If our signal is a 1D discrete time series, the AWGN vector added to the signal can be represented as $w∼N\left(0,\sigma^{2}I\right)$. To introduce masking noise into x, a certain fraction of the elements of x are randomly chosen and set to 0. Salt & pepper noise is when a certain fraction of the elements of x are randomly chosen and set to the min or max possible value. This is chosen by a fair coin flip. We can also convolve the input x with a Gaussian filter, blurring the input [18]. In the context of MNIST data set, we can corrupt the data by adding a block of white pixels to the center of the digits [18]. Salt & pepper noise and masking noise both corrupt a fraction of the elements in a signal significantly, while not affecting the others. By denoising, we are attempting to recover the original values of the elements that were corrupted. The only scenario where this is possible is if, in high dimensional probability distributions, there is a dependency between dimensions. What we expect when training the DAE is that it learns these dependencies. Thus, for low dimensional distribution, it does not make sense to use the DAE approach as much.

Convolutional Denoising Autoencoders (CDAEs) are DAEs with convolutional layers. Stacked denoising autoencoders (SAE) are when we are stacking layers of DAEs.

### 2.4. Bayesian Networks

For any joint probability distribution, their independence/dependence relationships can be depicted using Probabilistic Graphical Models. When the relationships are represented via directed acyclical graphs (DAGs), the graphical models are known as Bayesian Networks. Other names for Bayesian Networks include Directed Graphical Models and Belief Networks. To illustrate the use of Bayesian Networks, we will use an example from [19].

A woman named Tracey notices that her lawn is wet in the morning. She wonders whether it is from the rain or her accidentally leaving the sprinklers on the previous night. She then sees that her neighbor Jack also has a wet lawn. Her conclusion was that it rained last night.

Our variables are:

r∈{0,1}, where r=1 denotes it was raining last night, 0 denotes it was not raining last night.

s∈{0,1}, where s=1 denotes Tracey left the sprinklers on the previous night, and 0 otherwise.

j∈{0,1}, where j=1 indicates that Jack’s lawn is wet, and 0 otherwise.

t∈{0,1}, where t=1 Denotes that Tracey’s grass is wet, and 0 otherwise.

We can represent this with a joint probability function p(t,j,r,s). Using the chain rule of probability, we can decompose this into:

$$\begin{array}{cc} p(t,j,r,s) & =p(t|j,r,s)p(j,r,s)\\ & =p(t|j,r,s)p(j|r,s)p(r,s)\\ & =p(t|j,r,s)p(j|r,s)p(r|s)p(s) \end{array}$$

However, we can simplify this further by looking at the constraints. We know that the status of Tracey’s lawn does not depend on Jack’s; it depends on whether it rained and whether she left the sprinkler on. Thus, then:

p(t|j,r,s)=p(t|r,s)

We can also assume that the only variable affecting the status of Jack’s lawn is whether it was raining the night before. Thus, then:

p(j|r,s)=p(j|r)

We assume that the rain is affected by the sprinkler.

p(r|s)=p(r)

Thus, our simplified model is:

p(t,j,r,s)=p(t|j,r,s)p(j|r,s)p(r|s)p(s)=p(t|r,s)p(j|r)p(r)p(s)

**Figure 2:**
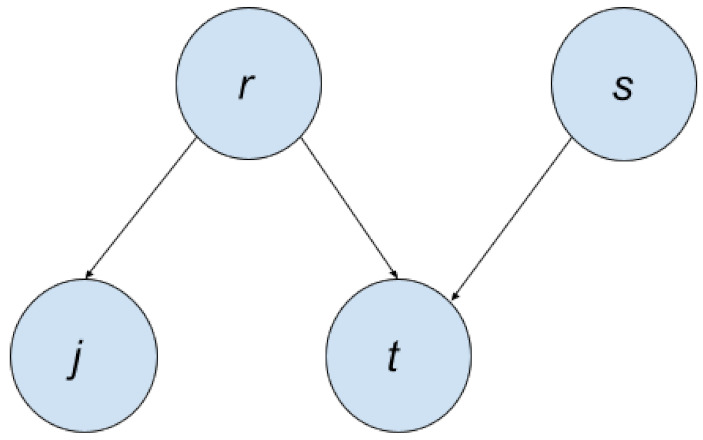
Bayesian Network of p(t|j,r,s). Since *r* affects *j* and *t*, it is a parent node of those two. *s* is also a parent node of *t*.

We can represent this with a Bayesian Network, as shown in Figure 2. Each node in this graph represents a variable from the joint distribution. Notice that there is a directed edge from *r* to *j*. This means that the *r* is the parent node of *j*, while *j* is the child node of the *r*. If any variable is a parent of another variable, it means it is on the right side of the conditional bar; like for p(j|r), *r* is on the right side of the conditional bar.

If we have a set of random variables $\left\{x_{1},\ldots ,x_{M}\right\}$ with certain conditional independence assumptions, we can represent their joint distribution as

(37) $$p_{\theta }\left(x_{1},\ldots ,x_{M}\right)=\prod_{j=1}^{M}p_{\theta }\left(x_{j}|Parents\left(x_{j}\right)\right)$$

Similarly, for a set of random vectors, $\left\{x_{1},\ldots ,x_{M}\right\}$ we can represent their joint distributions as:

(38) $$p_{\theta }\left(x_{1},\ldots ,x_{M}\right)=\prod_{j=1}^{M}p_{\theta }\left(x_{j}|Parents\left(x_{j}\right)\right)$$

For root nodes in the Bayesian Network, the set of parents is the empty set. Thus, they are marginal distributions. $Parents\left(x_{j}\right)$ denotes the set of parent variables for node $x_{j}$ in the Bayesian Network.

Initially, the parameterization of each conditional probability distribution was done with a lookup table or a linear model. In deep learning, we can use neural networks to parameterize conditional distributions; this is more flexible. The meaning of a neural network parameterizing a PDF is that it is part of the function that computes the PDF [20].

### 2.5. Generative Models vs. Discriminative Models

Given the PDF p(x,y), we generate a dataset $D={\left\{x^{\left(i\right)},y^{\left(i\right)}\right\}}_{i=1}^{N}$. We have the realizations of an i.i.d sequence $X={\left\{x^{\left(i\right)}\right\}}_{i=1}^{N}$ where $x^{\left(i\right)}\in R^{d}$ and each $x^{\left(i\right)}$ has a label $y^{\left(i\right)}$ associated with it [21]. A generative model would attempt to learn p(x,y). It would then generate new examples x from estimated distribution. The term p(y|x) would be a discriminative model; it attempts to estimate the label generating probability distribution function. A discriminative model can predict *y* given examples x, but it cannot generate a sample of x.

There are three types of generative models typically used in deep learning [22]: latent variable models, neural autoregressive models, and implicit models.

### 2.6. Latent Variable Models

**Figure 3:**
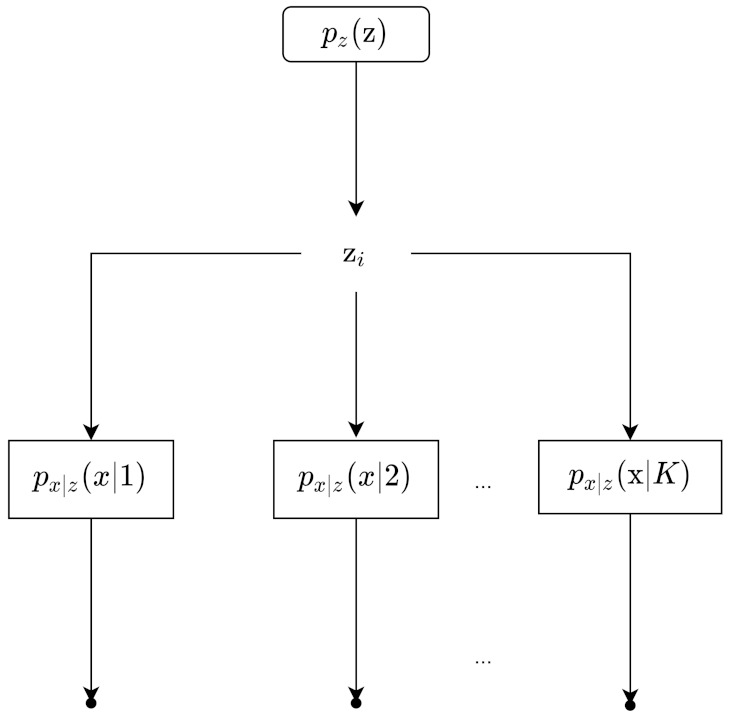
This shows the data generating process for a scenario with a latent variable. First, $z_{i}$ is sampled from p(z); we assume $z_{i}$ can take K values from 1 to K. Then, given $z_{i}$, we can generate *x*.

**Figure 4:**
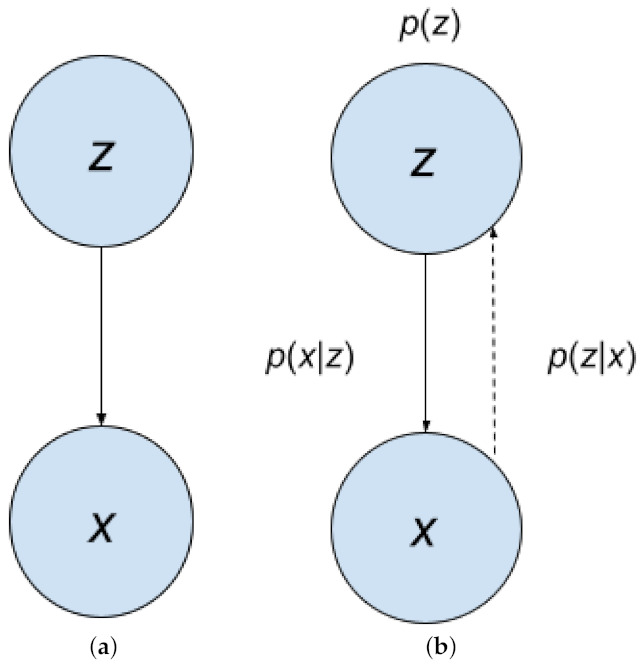
(**a**) A Bayesian Network representation of latent variable *z* and observation variable *x*. They can also be random vectors x and z; (**b**) another Bayesian Network for *z* and *x*, this time showing the dotted arrow for inference.

Latent variables are underlying variables; often times, they are not directly observable. An example given by Dr. Nuno Vasconcelos [23] is the bridge example. There is a bridge, and there are weight sensors measuring the weight of each car. However, we do not have a camera, so we do not know what type of car it is; it could be a compact, sedan, station wagon, pick up, or van. Thus, the hidden/latent variable, represented by random variable *z*, is the type of the car, and the observed variable is the weight measured, represented by random variable *x*. Figure 3 shows the process of data generation. Our example can be represented by the Bayesian Network in Figure 4a. The latent variables and observed variables can also be random vectors, denoted as z and x.

Sampling our observation takes two steps. First, a sample z comes from the probability distribution $p_{z}\left(z\right)$. Then, a sample x comes from $p_{x|z}\left(x|z\right)$. This is also called generation, represented by z→x in Figure 4a,b; it is represented by the solid arrow. With $p_{z}\left(z\right)$ with $p_{x|z}\left(x|z\right)$, we can obtain the joint density $p_{\left(x,z\right)}\left(x,z\right)$. Then, by marginalizing the joint density, we get $p_{x}\left(x\right)$. Then, from there, we can get p(z|x). Obtaining p(z|x) is represented by x→z. It is the inverse of generation, and is called inference. In Figure 4b, inference is represented by the dotted arrow. Inference can be obtained using Bayes Rule:

(39) $$p_{z|x}\left(z|x\right)=\frac{p_{x|z}\left(x|z\right)p_{z}\left(z\right)}{p_{x}\left(x\right)}$$

Often times, calculating $p_{x}\left(x\right)$ is intractable, making inference intractable through this method. This leads to approximation methods. Markov Chain Monte Carlo (MCMC) methods [13] are a common collection of methods [24] used for this. However, variational inference is a quicker family of methods. They do not guarantee that we will create exact samples from the target density function asymptotically like MCMC methods [25].

If we parameterize our model with θ, we use $p_{\theta }\left(x\right)$; this means that the PDF p(x) is associated with random variable *x* and has parameters represented by θ. We would attempt to learn θ using maximum likelihood estimation.

When we have a latent variable model $p_{\theta }\left(x,z\right)$, where a deep neural network parameterizes $p_{\theta }\left(x,z\right)$, it is called a deep latent variable model (DLVM) [20].

An example of a DLVM is given by the following [26]:

$$\begin{array}{c} z=\left(z_{1},z_{2},\cdots ,z_{K}\right)∼p\left(z;\beta \right)=\prod_{k=1}^{K}\beta_{k}^{z_{k}}{\left(1-\beta_{k}\right)}^{1-z_{k}}\\ x=\left(x_{1},x_{2},\cdots ,x_{L}\right)∼p_{\theta }\left(x|z\right)\Leftrightarrow \mathrm{Bernoulli}\left(x_{i};\mathrm{DNN}\left(z\right)\right) \end{array}$$

We have a random vector z, of length K, sampled from a multivariate Bernoulli distribution. This is then fed into a neural network, denoted by DNN, which outputs random vector x. The neural network can have L output units with sigmoid activations.

Another example of DLVM is the following [27]: If the observation data x of size L is binary data, the latent space is Gaussian latent space, and the observation model is a factorized multivariate Bernoulli distribution, we have the following formulas:

$$\begin{array}{cc} p(z) & =N(z;0,I)\\ a & ={\mathrm{DNN}}_{\theta }\left(z\right)\\ \log(p(x|z)) & =\sum_{j=1}^{L}\log\left(p\left(x_{j}|z\right)\right)=\sum_{j=1}^{L}\log\left(\mathrm{Bernoulli}\left(x_{j};a_{j}\right)\right)\\ & =\sum_{j=1}^{L}x_{j}\log\left(a_{j}\right)+\left(1-x_{j}\right)\log\left(1-a_{j}\right) \end{array}$$

where $a_{j}$ is a value between 0 and 1 and a is a vector with $a_{j}$’s; it can be implemented by having the output layer of the neural network have sigmoid activation functions.

A third example of a DLVM is where z is a Gaussian distribution, and p(x|z) can be a neural network with a softmax activation function for its output layer [26]. Our generative model in the case of latent variable models learns the joint PDF $p_{\theta }\left(z,x\right)$.

Overall, latent variable training can be summarized by the following four steps [26]:

(1) Sampling
  $z∼p_{z}\left(z\right)$
  $x∼p_{\theta }\left(x|z\right)$
(2) Evaluate likelihood $p_{\theta }\left(x\right)=\sum_{z}p_{z}\left(z\right)p_{\theta }\left(x|z\right)$
(3) Train $\underset{\theta }{\arg\max}\sum_{i=1}^{N}\log p_{\theta }\left(x^{\left(i\right)}\right)=\sum_{i}\log\sum_{z}p_{z}\left(z\right)p_{\theta }\left(x^{\left(i\right)}|z\right)$
(4) Representation x→z

Common latent variable models in deep learning include energy-based models, variational autoencoders, and flow models [28,29]. The VAE explicitly models the density of the distribution, so it has a prescribed Bayesian Network.

### 2.7. Neural Autoregressive Models

In time series analysis, an autoregressive model of order p is denoted as AR(p) [30]. If we have a time series {y[n],y[n−1],…,y[n−p]}, it is an AR(p) process if it satisfies the following equation:

(40) $$y\left[n\right]=\sum_{j=1}^{p}a_{j}y\left[n-j\right]+w\left[n\right]+\mu$$

y[n] denotes the value of *y* (a scalar value) at time n. y[n] is a linear combination of the p past values of *y*, weighted by scalar coefficients $a_{j}$ plus some white noise w[n] and the mean μ of the process (E[y[n]]=μ).

In neural networks, there is a subtype of the AR model, called the neural autoregressive model or autoregressive neural density estimators; in deep learning literature, this subtype is often just called an autoregressive model. The neural autoregressive model involves using a Bayesian Network structure where the conditional probabilities are set to neural networks.

If we are given a Bayesian Network representation for a model, we can get a tractable gradient for our log likelihood by setting the conditional probability distributions to neural networks [31]:

(41) $$\log\left(p_{\theta }\left(x\right)\right)=\sum_{i=1}^{d}\log\left(p_{\theta }\left(x_{i}|Parents\left(x_{i}\right)\right)\right)$$

Parent denotes the parent nodes of $x_{i}$. If we assume that our Bayesian Network is fully expressive, any joint probability distribution can be decomposed to a product of conditionals, using the probability chain rule and conditional independence assumptions:

(42) $$\log\left(p\left(x\right)\right)=\sum_{i=1}^{d}\log\left(p\left(x_{i}|x_{1:i-1}\right)\right)$$

This is called a neural autoregressive model. Common neural autoregressive models include Neural Autoregressive Distribution Estimation (NADE) [32,33], Masked Autoencoder for Distribution Estimation (MADE) [34], Deep AutoRegressive Networks (DARN) [35], PixelRNN [36], PixelCNN [36,37], and WaveNet [38].

Neural AR models are slower because they sequentially generate from one dimension at a time. They also tend to model local structure better than global structure.

### 2.8. Generative Adversarial Networks (GANs)

Two major implicit models are Generative Stochastic Networks (GSNs) and GANs [39,40].

A GAN trains a generative model Gen and a discriminative model Dis simultaneously. Gen attempts to estimate the distribution of the data, while Dis tries to estimate the probability that the data came from the training set rather than Gen. Gen tries to maximize the probability that the discriminator makes a mistake. Typically for a GAN, Dis is only used during training, but, afterwards, it is discarded.

The Bayesian Network in Figure 4a from Section 2.6 can also represent the generator from a GAN. This is because a random noise term *z*, often sampled from a uniform distribution, is the input to Gen, which outputs synthetic data Gen(z); we can also write Gen(z) as $\hat{x}$. The GAN implicitly models the distribution and so it has an implicit Bayesian network [41]. Thus, the GAN is both an implicit model and has a latent variable model.

**Figure 5:**
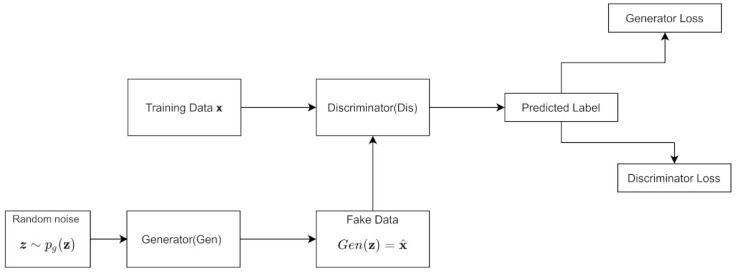
GAN architecture. A random noise vector drives our generator to create fake data. The fake data and training data are sent to the discriminator, which attempts to classify which data are fake or real.

The algorithm is outlined in Algorithm 1. The noise prior $p_{g}\left(z\right)$ is the input to the generator; it is often typically a uniform distribution. The data generating distribution is denoted $p_{d\;}\left(x\right)$; it is the data behind our real distribution. The hyperparameter k denotes how many steps the discriminator is applied. $\theta_{G}$ represents the weights and biases of Gen, while $\theta_{D}$ represents the weights and biases of Dis; the subscripts denote their association with the discriminator and generator. Figure 5 shows the architecture.
**Algorithm 1:** Original GAN algorithm**for**do# of training iterations:    **for** k **do** steps        Sample minibatch $\left\{z^{\left(1\right)},\ldots ,z^{\left(m\right)}\right\}$∼$p_{g}\left(z\right)$        Sample minibatch $\left\{x^{\left(1\right)},\ldots ,x^{\left(m\right)}\right\}$∼$p_{d\;}\left(x\right)$        Update the weights of the discriminator by ascending its stochastic gradient:
$$\nabla_{\theta_{D}}\frac{1}{m}\sum_{i=1}^{m}\left[\log\left(Dis\left(x^{\left(i\right)}\right)\right)+\log\left(1-Dis\left(Gen\left(z^{\left(i\right)}\right)\right)\right)\right]$$    **end for**    Sample minibatch $\left\{z^{\left(1\right)},\ldots ,z^{\left(m\right)}\right\}$∼$p_{g}\left(z\right)$    Update the weights of the generator by descending its stochastic gradient:
$$\nabla_{\theta_{G}}\frac{1}{m}\sum_{i=1}^{m}\log\left(1-Dis\left(Gen\left(z^{\left(i\right)}\right)\right)\right).$$**end for**

Gradient updates can use any rule. The loss function is similar to JS Divergence. When Dis is optimal, the weights of Gen are updated in a way that it minimizes the JS Divergence.

The original GAN had issues such as mode collapse, convergence, and vanishing gradients. The Wasserstein GAN [42] is a class of GAN meant to improve on these flaws; it uses Wasserstein distance instead. Conditional GANs (CGANs) [43] are another type of GAN that attempts to alleviate some of the flaws of the GAN.

The Deep Convolutional GAN (DCGAN) [44] is what many GANs are based on; it uses ADAM to optimize, uses an all convolutional network [45], and has batch normalization [46] in most layers for Dis and Gen. Gen’s last and Dis’s first layer are not batch normalized. Other GAN methods include Periodic Spatial GAN (PSGAN) [47], INFOGAN [48], CycleGAN [49], StyleGAN [50], and Self-Attention GAN (SAGAN) [51].

### 2.9. Gradient Estimation

#### 2.9.1. REINFORCE Estimator/Score Function

Two important gradient estimators are the score function and the pathwise gradient estimator [22,26,52,53]. The score function (also known as the REINFORCE estimator) can handle non-differentiable functions; the downside is that it has a high variance.

If you have a function $p_{\theta }\left(x\right)$, which is a PDF of random variable x parameterized by θ, then the score function is $\nabla_{\theta }\log\left(p_{\theta }\left(x\right)\right)$. This is the derivative of the log of our PDF w.r.t to θ. This score function can be written as:

(43) $$\nabla_{\theta }\log\left(p_{\theta }\left(x\right)\right)=\frac{\nabla_{\theta }p_{\theta }\left(x\right)}{p_{\theta }\left(x\right)}$$

The score function’s expectation is zero:

$$E_{p_{\theta }\left(x\right)}\left[\nabla_{\theta }\log\left(p_{\theta }\left(x\right)\right)\right]=\int p_{\theta }\left(x\right)\frac{\nabla_{\theta }p_{\theta }\left(x\right)}{p_{\theta }\left(x\right)}dx=\nabla_{\theta }\int p_{\theta }\left(x\right)dx=\nabla_{\theta }1=0$$

The score function’s variance is the Fisher information. The estimator for the score function can be derived as follows:

(44) $$\begin{array}{cc} \; & \nabla_{\theta }E_{p_{\theta }\left(x\right)}\left[f\left(x\right)\right]=\nabla_{\theta }\int p_{\theta }\left(x\right)f\left(x\right)dx=\int f\left(x\right)\nabla_{\theta }p_{\theta }\left(x\right)dx\\ \; & =\int p_{\theta }\left(x\right)f\left(x\right)\nabla_{\theta }\log\left(p_{\theta }\left(x\right)\right)dx\\ \; & =E_{p_{\theta }\left(x\right)}\left[f\left(x\right)\nabla_{\theta }\log\left(p_{\theta }\left(x\right)\right)\right]\\ \; & \approx \frac{1}{L}\sum_{l=1}^{L}f\left(x^{\left(l\right)}\right)\nabla_{\theta }\log\left(p_{\theta }\left(x^{\left(l\right)}\right)\right);\;x^{\left(l\right)}∼p_{\theta }\left(x\right) \end{array}$$

#### 2.9.2. Pathwise Gradient Estimator

The pathwise gradient estimator is also known as the reparameterization trick or the pathwise derivative estimator. However, it has low variance, so it is a common choice. More details about this estimator will be shown in the next section. For a continuous distribution for x, direct sampling has an equivalent indirect process:

$$x∼p_{\theta }\left(x\right)\;\equiv \;x=g\left(\epsilon ,\theta \right),\;\epsilon ∼p\left(\epsilon \right)$$

This statement means an indirect way to create samples x from $p_{\theta }\left(x\right)$ is to sample from p(ϵ) first; this distribution is independent of θ. The next step is to apply a transformation with g(ϵ,θ) which is deterministic. This can be called a sampling path or a sampling process.

For indirect sampling from a Gaussian distribution denoted by N(x;μ,C), we can reparameterize it by making g(ϵ,θ) a location scale transformation, given by $g\left(\epsilon ,\theta \right)=\mu +L\epsilon ,\;\mathrm{where}\;{\mathrm{LL}}^{T}=C.\;$L is a lower triangular matrix with nonzero diagonal values and ϵ is sampled from a standard isotropic multivariate Gaussian p(ϵ)=N(0,I).

We can derive the gradient estimator by the following:

$$\begin{array}{cc} & \nabla_{\theta }E_{p_{\theta }\left(x\right)}\left[f\left(x\right)\right]=\nabla_{\theta }\int p_{\theta }\left(x\right)f\left(x\right)dx\\ & =\nabla_{\theta }\int p\left(\epsilon \right)f\left(g\left(\epsilon ,\theta \right)\right)d\epsilon \\ & =E_{p(\epsilon )}\left[\nabla_{\theta }f\left(g\left(\epsilon ,\theta \right)\right)\right]\\ & \approx \frac{1}{L}\sum_{l=1}^{L}\nabla_{\theta }f\left(g\left(\epsilon^{\left(l\right)},\theta \right)\right);\;\epsilon^{\left(l\right)}∼p\left(\epsilon \right) \end{array}$$

Thus, our pathwise gradient estimator where our x is distributed according to N(x;μ,C) is given by:

(45) $$\begin{array}{c} \nabla_{\theta }E_{p_{\theta }\left(x\right)}\left[f\left(x\right)\right]\approx \frac{1}{L}\sum_{l=1}^{L}\nabla_{\theta }f\left(g\left(\epsilon^{\left(l\right)},\theta \right)\right);\;\epsilon^{\left(l\right)}∼p\left(\epsilon \right) \end{array}$$

where $g\left(\epsilon ,\theta \right)=\mu +L\epsilon ,{\mathrm{LL}}^{T}=C,p\left(\epsilon \right)=N\left(0,I\right)$.

## 3. Variational Inference

In Bayesian statistics, parameters that we estimate are random variables instead of deterministic variables. For latent random vector z and observation variables/vector x,$p_{\theta }\left(z|x\right)$ is known as the posterior distribution; p(z) is the prior distribution, $p_{\theta }\left(x\right)$ is the model evidence or marginal likelihood, and $p_{\theta }\left(x|z\right)$ is the likelihood. We perform updates on prior $p_{z}\left(z\right)$ using Bayesian rule.

Variational inference is a particular method for approximating the posterior distribution. We approximate the posterior distribution $p_{\theta }\left(z|x\right)$ with the approximate posterior $q_{\phi }\left(z\right)$; it is also known as the variational posterior, where ϕ represents the variational parameters. We will optimize over ϕ so we can fit the variational posterior to the real posterior. Any valid distribution for $q_{\phi }\left(z\right)$ can be used as long as we can sample data from it and we can compute $\log(q_{\phi }\left(z\right))$ and $\nabla_{\phi }\log\left(q_{\phi }\left(z\right)\right)$. Thus, we want to solve the following for all $x^{\left(i\right)}$

(46) $$\min_{q_{\phi }\left(z\right)}D_{KL}\left(q_{\phi }\left(z\right)∥p_{\theta }\left(z|x^{\left(i\right)}\right)\right)$$

We are taking the reverse-KL Divergence between *p* and *q*. There are different posteriors for each datapoint $x^{\left(i\right)}$, so we learn a different ϕ for each datapoint. To make this calculation more quick, we can use an amortized formulation for variational inference:

(47) $$\min_{\phi }\sum_{i}D_{KL}\left(q_{\phi }\left(z|x^{\left(i\right)}\right)∥p_{\theta }\left(z|x^{\left(i\right)}\right)\right)$$

In this formulation, we predict ϕ with a neural network, called an inference network; variational parameters ϕ refer to the parameters of this inference network. The downside of this formulation is less precision. With this, we can derive the Evidence Lower Bound (ELBO):

$$\begin{array}{ccc} & D_{KL}\left(q_{\phi }\left(z|x\right)∥p_{\theta }\left(z|x\right)\right)=\int_{-\infty }^{\infty }q_{\phi }\left(z|x\right)\log\left(\frac{q_{\phi }\left(z|x\right)}{p_{\theta }\left(z|x\right)}\right)dz= & E_{q_{\phi }\left(z|x\right)}\left[\log\left(\frac{q_{\phi }\left(z|x\right)}{p_{\theta }\left(z|x\right)}\right)\right] \end{array}$$

$$\begin{array}{cc} & =E_{q_{\phi }\left(z|x\right)}\left[\log\left(q_{\phi }\left(z|x\right)\right)-\log\left(p_{\theta }\left(z|x\right)\right)\right]=E_{q_{\phi }\left(z|x\right)}\left[\log\left(q_{\phi }\left(z|x\right)\right)-\log\left(\frac{p_{\theta }\left(x|z\right)p\left(z\right)}{p_{\theta }\left(x\right)}\right)\right]\\ & =E_{q_{\phi }\left(z|x\right)}\left[\log\left(q_{\phi }\left(z|x\right)\right)-\log\left(p_{\theta }\left(x|z\right)\right)-\log\left(p\left(z\right)\right)+\log\left(p_{\theta }\left(x\right)\right)\right]\\ & =E_{q_{\phi }\left(z|x\right)}\left[\log\left(q_{\phi }\left(z|x\right)\right)-\log\left(p_{\theta }\left(x|z\right)\right)-\log\left(p\left(z\right)\right)\right]+\log\left(p_{\theta }\left(x\right)\right)\\ & =-L\left(\theta ,\phi ;x\right)+\log(p_{\theta }\left(x\right)) \end{array}$$

$\mathrm{where}\;L\left(\theta ,\phi ;x\right)=E_{q_{\phi }\left(z|x\right)}\left[-\log\left(q_{\phi }\left(z|x\right)\right)+\log\left(p_{\theta }\left(x|z\right)\right)+\log\left(p\left(z\right)\right)\right]$.

Since $D_{KL}(\left(q_{\phi }\left(z|x\right)∥p_{\theta }\left(z|x\right)\right)\geq 0,\mathrm{we}\;\mathrm{have}-L\left(\theta ,\phi ;x\right)+\log\left(p_{\theta }\left(x\right)\right)\geq 0$.

Thus, we arrive at:

(48) $$L\left(\theta ,\phi ;x\right)\leq \log(p_{\theta }\left(x\right))$$

or

(49) $$\begin{array}{ccc} L\left(\theta ,\phi ;x\right)=E_{q_{\phi }\left(z|x\right)}\left[-\log\left(q_{\phi }\left(z|x\right)\right)+\log\left(p_{\theta }\left(x|z\right)\right)+\log\left(p\left(z\right)\right)\right] & & \\ \leq \log(p_{\theta }\left(x\right)) \end{array}$$

This is called the Evidence Lower Bound (ELBO), or variational lower bound.

We can derive a second formulation as follows:

$$\begin{array}{cc} & L\left(\theta ,\phi ;x\right)=E_{q_{\phi }\left(z|x\right)}\left[-\log\left(q_{\phi }\left(z|x\right)\right)+\log\left(p_{\theta }\left(x|z\right)\right)+\log\left(p\left(z\right)\right)\right]=\\ & E_{q_{\phi }\left(z|x\right)}\left[\log\left(\frac{p_{\theta }\left(x,z\right)}{q_{\phi }\left(z|x\right)}\right)\right]\leq \log\left(p_{\theta }\left(x\right)\right) \end{array}$$

Thus, we arrive at:

(50) $$L\left(\theta ,\phi ;x\right)=E_{q_{\phi }\left(z|x\right)}\left[\log\left(\frac{p_{\theta }\left(x,z\right)}{q_{\phi }\left(z|x\right)}\right)\right]\leq \log\left(p_{\theta }\left(x\right)\right)$$

We also derive a third formulation as follows:

$E_{q_{\phi }\left(z|x\right)}\left[-\log\left(q_{\phi }\left(z|x\right)\right)+\log\left(p_{\theta }\left(x|z\right)\right)+\log\left(p\left(z\right)\right)\right]$

$\left.=E_{q_{\phi }\left(z|x\right)}\left[\log\left(p_{\theta }\left(x|z\right)\right)\right]+E_{q_{\phi }\left(z|x\right)}\left[\log\left(\frac{p(z)}{q_{\phi }\left(z|x\right)}\right)\right]\right.$

$=E_{q_{\phi }\left(z|x\right)}\left[\log\left(p_{\theta }\left(x|z\right)\right)\right]-D_{KL}\left(q_{\phi }\left(z|x\right)∥p\left(z\right)\right)$

Thus, we arrive at:

(51) $$L\left(\theta ,\phi ;x\right)=E_{q_{\phi }\left(z|x\right)}\left[\log\left(p_{\theta }\left(x|z\right)\right)\right]-D_{KL}\left(q_{\phi }\left(z|x\right)∥p\left(z\right)\right)\leq \log\left(p_{\theta }\left(x\right)\right)$$

We would train our model by maximizing the ELBO L(θ,ϕ;x) with respect to θ and ϕ, which are our model parameters and variational parameters.

Taking the gradient of ELBO w.r.t. to θ is easily calculated.

$\nabla_{\theta }L\left(\theta ,\phi ;x\right)=\nabla_{\theta }E_{q_{\phi }\left(z|x\right)}\left[\log\left(\frac{p_{\theta }\left(x,z\right)}{q_{\phi }\left(z|x\right)}\right)\right]$

$=E_{q_{\phi }\left(z|x\right)}\left[\nabla_{\theta }\log(p_{\theta }\left(x,z\right)\right)]$

$\approx \frac{1}{L}\sum_{l=1}^{L}\nabla_{\theta }\log\left(p_{\theta }\left(x,z^{\left(l\right)}\right)\right)\;\mathrm{with}\;z^{\left(l\right)}∼q_{\phi }\left(z|x\right)$

Thus, we estimate the gradient w.r.t to θ using the formula

(52) $$\frac{1}{L}\sum_{l=1}^{L}\nabla_{\theta }\log\left(p_{\theta }\left(x,z^{\left(l\right)}\right)\right)\;\mathrm{with}\;z^{\left(l\right)}∼q_{\phi }\left(z|x\right)$$

We can generate samples from *q* and use it to calculate each $\nabla_{\theta }\log\left(p_{\theta }\left(x,z^{\left(l\right)}\right)\right)$, and average these individual gradients to estimate the gradient of the ELBO w.r.t θ.

Estimating $\nabla_{\phi }L\left(\theta ,\phi ;x\right)$ is more difficult. This is because we cannot bring the gradient inside the expectation because the expectation is a function of ϕ.

$\nabla_{\phi }L\left(\theta ,\phi ;x\right)=\nabla_{\phi }E_{q_{\phi }\left(z|x\right)}\left[\log\left(\frac{p_{\theta }\left(x,z\right)}{q_{\phi }\left(z|x\right)}\right)\right]\neq E_{q_{\phi }\left(z|x\right)}\left[\nabla_{\phi }\left(\log\left(p_{\theta }\left(x,z\right)\right)-\log\left(q_{\phi }\left(z|x\right)\right)\right)\right]$

The score function and pathwise gradient estimator are both possible methods to estimate the gradient. The score function can apply to latent variables that are continuous or discrete. The pathwise gradient estimator applied to continuous latent variables and requires that the function being estimated is differentiable. For the pathwise gradient estimator, we reparameterize a sample from $q_{\phi }\left(z|x\right)$ by expressing it as a function of a sample ϵ from some fixed distribution p(ϵ):

(53) z=g(ϵ,ϕ,x)

p(ϵ) is independent of x or ϕ. We can bring $\nabla_{\phi }$ inside the expectation because p(ϵ) does not depend on ϕ. We assume g(ϵ,ϕ,x) is differentiable with respect to ϕ. Thus, our pathwise gradient estimator for $\nabla_{\phi }L\left(\theta ,\phi ;x\right)$, where z is supposed to be sampled from N(z;μ,C), can be derived using Equation (45):

(54) $$\begin{array}{ccc} \nabla_{\phi }E_{q_{\phi }\left(z\right)}\left[f\left(z\right)\right]\approx \frac{1}{L}\sum_{l=1}^{L}\nabla_{\phi }f\left(g\left(\epsilon^{\left(l\right)};\phi \right)\right);\;\epsilon^{\left(l\right)}∼p\left(\epsilon \right) & & \\ \mathrm{where}\;z=g\left(\epsilon ,\phi ,x\right)=\mu +L\epsilon ,LL^{T}=C,p\left(\epsilon \right)=N\left(0,I\right) \end{array}$$

We can choose our $q_{\phi }\left(z|x\right)=N\left(z;\mu ,\mathrm{diag}\left(\sigma^{2}\right)\right)$, which is a multivariate Gaussian distribution with a diagonal matrix as its covariance matrix. μ is the mean vector and $\sigma^{2}$ is a vector that creates covariance matrix $\mathrm{diag}\left(\sigma^{2}\right)$, which we can sample using

(55) z=g(ϵ,ϕ,x)=μ+Lϵ=μ+σ⊙ϵ

where $\mathrm{diag}\left(\sigma {}^{2}\right)=LL^{T}$, and ⊙ is an elementwise multiplication operator. If we have $z∼N\left(\mu ,\sigma^{2}\right)$, we can reparameterize it by

$$z=\mu +\sigma \epsilon ,\epsilon ∼p\left(\epsilon \right)=N\left(0,1\right)$$

If we are not using amortized variational inference, the formula for ELBO is different.

## 4. The Variational Autoencoder

The Variational Autoencoder uses an inference network as its encoder. The VAE has a MLP encoder and an MLP decoder [2]. The encoder is the variational posterior $q_{\phi }\left(z|x\right)$ and is an inference/recognition model. The decoder is a generative model, and it represents the likelihood $p_{\theta }\left(x|z\right)$.

A joint inference distribution can be defined as

$$q_{\phi }\left(x,z\right)\equiv p_{\theta }\left(x\right)q_{\phi }\left(z|x\right)$$

$q_{\phi }\left(z\right)$ is called the aggregated posterior, given by the following:

(56) $$\int_{range(x)}p_{\theta }\left(x\right)q_{\phi }\left(z|x\right)dx\equiv \int_{range(x)}q_{\phi }\left(x,z\right)dx\equiv q_{\phi }\left(z\right)$$

The prior is a standard multivariate isotropic Gaussian distribution, $p_{z}\left(z\right)=N\left(z;0,I\right)$, while the likelihood function $p_{\theta }\left(x|z\right)$ is a Gaussian distribution or a Bernoulli distribution. The Gaussian likelihood is $p_{\theta }\left(x|z\right)=N\left(x;\mu_{decoder},diag\left(\sigma_{decoder}^{2}\right)\right)$, which is a multivariate Gaussian with a diagonal covariance matrix. The posterior distribution can be any PDF, but is assumed to be approximately a multivariate Gaussian with a diagonal covariance matrix. The variational posterior is also taken to be a multivariate Gaussian with a diagonal covariance matrix, given by $q_{\phi }\left(z|x\right)=N\left(z;\mu ,\mathrm{diag}\left(\sigma^{2}\right)\right)$; μ and $\sigma^{2}$ from the variational posterior are outputs of the encoder.

The weights and biases for the encoder are the variational parameters ϕ, while the weights and biases for the decoder are the model parameters θ.

We sample z from the encoder using the reparameterization trick; so the encoder outputs μ and $\sigma^{2}$, and generates ϵ from N(0,I). The variable z is the input to the decoder, which then generates a new example of x. Equation (55) is used.

Note that, in practice, the encoder outputs log($\sigma^{2}$) instead of $\sigma^{2}$ to ensure positive values for $\sigma^{2}$. We can then retrieve $\sigma^{2}$ by taking the exponential of log($\sigma^{2}$). We can also retrieve σ by taking the exponential of (0.5)log($\sigma^{2}$). In the literature, they often refer to the output as σ instead of $\sigma^{2}$.

If we assume that our encoder has one hidden layer, and the distribution is multivariate Gaussian with a diagonal covariance matrix, we can write it and the sampling process as:

$$\begin{array}{c} h=\tanh\left(W_{1}x+b_{1}\right)\\ \mu =W_{2}h+b_{2}\\ \log\left(\sigma^{2}\right)=W_{3}h+b_{3}\\ z=\mu +\sigma ⊙\epsilon \end{array}$$

$\left\{W_{1},W_{2},W_{3},b_{1},b_{2},b_{3}\right\}$ are the weights and biases of the encoder MLP, so they are variational parameters ϕ.

An encoder with any number of hidden layers can be summarized with the following equations:

$$\begin{array}{cc} \epsilon & ∼N(0,I)\\ \left(\mu ,\log\left(\sigma^{2}\right)\right) & =\;\mathrm{EncoderNeuralNet}{\;}_{\phi }\left(x\right)\\ z & =\mu +\sigma ⊙\epsilon \end{array}$$

For the decoder, we have two choices.

(1) Multivariate Bernoulli MLP for decoder:

The likelihood $p_{\theta }\left(x|z\right)$ is a multivariate Bernoulli. With decoder input z, the probabilities of the decoder are calculated with the MLP. $\left\{W_{1},W_{2},b_{1},b_{2}\right\}$ are the weights and biases of the decoder MLP. The hidden layer has a tanh activation function, while the output layer has a sigmoid activation function sig(.). The output is plugged into the log likelihood, getting a Cross-Entropy (CE) function.

$$\begin{array}{cc} h= & \tanh\left(W_{1}z+b_{1}\right)\\ y= & sig\left(W_{2}h+b_{2}\right)\\ \log(p(x|z)) & =\sum_{i=1}^{D}x_{i}\log\left(y_{i}\right)+\left(1-x_{i}\right)\cdot \log\left(1-y_{i}\right) \end{array}$$

The equations for more hidden layers can be written as

$$\begin{array}{cc} y & =\;\mathrm{DecoderNeuralNet}{\;}_{\theta }\left(z\right)\\ \log(p(x|z)) & =\sum_{j=1}^{d}\log\left(p\left(x_{j}|z\right)\right)=\sum_{j=1}^{d}\log\left(\mathrm{Bernoulli}\left(x_{j};y_{j}\right)\right)\\ & =\sum_{j=1}^{d}\left(x_{j}\log\left(y_{j}\right)+\left(1-x_{j}\right)\log\left(1-y_{j}\right)\right) \end{array}$$

The variable d is the dimensionality of x and Bernoulli (.;y) is the Bernoulli PMF. $\forall y_{j}\in y:0\leq y_{j}\leq 1$. This y can be implemented by making the last layer of the decoder a sigmoid function. This is similar to the second example of a DLVM in Section 2.6.

(2) Gaussian MLP as decoder

This is the case where the decoder distribution is a multivariate Gaussian with a diagonal covariance structure:

$$\begin{array}{cc} h & =\tanh\left(W_{3}z+b_{3}\right)\\ \mu_{decoder} & =W_{4}h+b_{4}\\ \log(\sigma_{decoder}^{2}) & =W_{5}h+b_{5}\\ \log(p(x|z)) & =\log(N\left(x;\mu ,diag(\sigma_{decoder}^{2})\right))\\ & =\frac{-L}{2}log\left(2\pi \right)+\frac{-1}{2}\sum \sigma_{decoder,i}^{2}+\sum \frac{{\left(x_{i}-\mu_{decoder,i}\right)}^{2}}{-2\sigma_{decoder,i}^{2}} \end{array}$$

where $\left\{W_{3},W_{4},W_{5},b_{3},b_{4},b_{5}\right\}$ are the weights and biases of the decoder MLP, so they are the model parameters θ.

**Figure 6:**
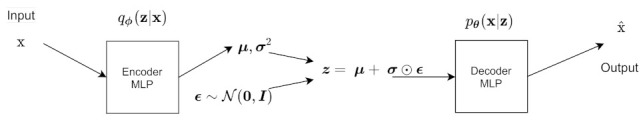
Diagram of the variational autoencoder. The input x goes into the encoder, which then creates a sample of z using the reparameterization trick. z is the input to the decoder, which outputs a new example of x, denoted as $\tilde{x}$.

Those are the derivations for the forward propagation in the VAE. Figure 6 shows the architecture of the VAE for a forward pass, excluding the ELBO calculation.

We will get ELBO estimates by looking at this equation:

$$E_{q_{\phi }\left(z|x\right)}\left[\log\left(p_{\theta }\left(x|z\right)\right)\right]-D_{KL}\left(q_{\phi }\left(z|x\right)∥p_{z}\left(z\right)\right)\leq \log\left(p_{\theta }\left(x\right)\right)$$

$E_{q_{\phi }\left(z|x\right)}\left[\log\left(p_{\theta }\left(x|z\right)\right)\right]$ is a reconstruction loss, while $D_{KL}\left(q_{\phi }\left(z|x\right)∥p\left(z\right)\right)$ is a regularizing term.

We derive the expression to get $-D_{KL}\left(q_{\phi }\left(z|x\right)∥p_{z}\left(z\right)\right)$ [2].

$$\begin{array}{cc} & D_{KL}\left(q_{\phi }\left(z|x\right)∥p_{z}\left(z\right)\right)=\int q_{\phi }\left(z|x\right)\left(\log\left(p_{z}\left(z\right))-\log\left(q_{\phi }\left(z|x\right)\right))dz\right.\right.\\ & =\int q_{\phi }\left(z|x\right)(\log\left(p_{z}\left(z\right)\right)dz-\int q_{\phi }\left(z|x\right)\log\left(q_{\phi }\left(z|x\right)\right)dz \end{array}$$

Split into two parts:

$$\begin{array}{cc} & \int q_{\phi }\left(z|x\right)(\log\left(p_{z}\left(z\right)\right)dz=\int N\left(z;\mu ,C_{v}\right)\log\left(N\left(z;0,I\right)\right)dz\\ & =\frac{-j}{2}\log\left(2\pi \right)+\frac{1}{2}\sum_{j=1}^{J}\left(\mu_{j}^{2}+\sigma_{j}^{2}\right) \end{array}$$

$$\begin{array}{cc} & \int q_{\phi }\left(z|x\right)\log\left(q_{\phi }\left(z|x\right)\right)dz=\int N\left(z;\mu ,C_{v}\right)\log\left(N\left(z;\mu ,C_{v}\right)\right)dz\\ & =\frac{-j}{2}\log\left(2\pi \right)-\frac{1}{2}\sum_{j=1}^{J}\left(1+\log\left(\sigma_{j}^{2}\right)\right) \end{array}$$

We can then add the two terms:

$$\begin{array}{cc} & -D_{KL}\left(q_{\phi }\left(z|x\right)∥p_{z}\left(z\right)\right)=\int q_{\phi }\left(z|x\right)\log\left(q_{\phi }\left(z|x\right)\right)dz-\int q_{\phi }\left(z|x\right)(\log\left(p_{z}\left(z\right)\right)dz.\\ & =\frac{-j}{2}\log\left(2\pi \right)-\frac{1}{2}\sum_{j=1}^{J}\left(1+\log\left(\sigma_{j}^{2}\right)\right)-\left(\frac{-j}{2}\log\left(2\pi \right)+\frac{1}{2}\sum_{j=1}^{J}\left(\mu_{j}^{2}+\sigma_{j}^{2}\right)\right)\\ & =\frac{1}{2}\sum_{j=1}^{J}\left(1+\log\left(\sigma_{j}^{2}\right)-\sigma_{j}^{2}-\mu_{j}^{2}\right) \end{array}$$

$\sigma_{j}^{2}$ and $\mu_{j}$ represent the jth component of the $\sigma^{2}$ and μ vectors for a given datapoint inputted into the encoder.

Our total training set is $X={\left\{x^{\left(i\right)}\right\}}_{i=1}^{N}$. Our Stochastic Gradient Variational Bayes Estimator ( SGVB Estimator) estimates the lower bound for one datapoint by:

$$\begin{array}{cc} & \tilde{L}\left(\theta ,\phi ;x^{\left(i\right)}\right)=-D_{KL}\left(q_{\phi }\left(z|x^{\left(i\right)}\right)∥p_{\theta }\left(z\right)\right)+\frac{1}{L}\sum_{l=1}^{L}\left(\log p_{\theta }\left(x^{\left(i\right)}|z^{\left(i,l\right)}\right)\right)\\ & \;\mathrm{where}\;z^{\left(i,l\right)}=g\left(\phi ,\epsilon^{\left(i,l\right)},x^{\left(i\right)}\right)\;\mathrm{and}\;\epsilon^{\left(l\right)}∼p\left(\epsilon \right) \end{array}$$

We can then have our SGVB estimator using minibatches from X, where $X^{\left(M\right)}$ is the M-th minibatch.

We can have $X^{\left(M\right)}={\left\{x^{\left(i\right)}\right\}}_{i=1}^{M}$, where $X^{\left(M\right)}$ is a minibatch from X with M datapoints. Then, we can estimate the ELBO over the full dataset X, denoted by L(θ,ϕ;X):

(57) $$L\left(\theta ,\phi ;X\right)≃{\tilde{L}}^{\left(M\right)}\left(\theta ,\phi ;X^{\left(M\right)}\right)=\frac{N}{M}\sum_{i=1}^{M}\tilde{L}\left(\theta ,\phi ;x^{\left(i\right)}\right)$$

where ${\tilde{L}}^{\left(M\right)}\left(\theta ,\phi ;X^{\left(M\right)}\right)$ is a minibatch estimator. Empirically, it has been shown that L could be set to 1 if the size of the minibatch is large.

The method used to train the VAE to find the parameters is called the Auto-Encoding Variational Bayes (AEVB) algorithm. Algorithm 2 shows the steps of the AEVB algorithm.
**Algorithm 2:** AEVB algorithm using minibatchesθ,ϕ←Initializeparameters**for**do# of training iterations:    $X^{\left(M\right)}\leftarrow \;\mathrm{Random}\;\mathrm{minibatch}\;\mathrm{of}\;M\;\mathrm{datapoints}\;\mathrm{from}\;\mathrm{full}\;\mathrm{dataset}\;X$    Sampleϵ∼p(ϵ)    $\mathrm{Calculate}\;\mathrm{minibatch}\;\mathrm{estimate}\;{\tilde{L}}^{\left(M\right)}\left(\theta ,\phi ;X^{\left(M\right)},\epsilon \right)$    $\mathrm{Calculate}\;\mathrm{gradients}\;\mathrm{of}\;\mathrm{minibatch}\;\mathrm{estimator}\;\nabla_{\theta ,\phi }{\tilde{L}}^{\left(M\right)}\left(\theta ,\phi ;X^{\left(M\right)},\epsilon \right)$    θ,ϕ←UpdateparametersusinggradientswithmethodslikeSGDorAdagrad**end for**

We have presented and derived the original variational autoencoder model; however, the variational autoencoder often refers more of a general framework, where we can choose different prior, posterior and likelihood distributions, along with many other variations. Thus, the VAE framework can refer to a continuous latent variable deep learning model that uses the reparameterization trick and amortized variational inference [22].

## 5. Problems/Tradeoffs with the VAE

While being a powerful model, the VAE has multiple problems and trade-offs. We will cover variance loss, image blurriness, posterior collapse, disentanglement, the balancing issue, the origin gravity effect, and the curse of dimensionality. We then compare the VAE with the GAN.

### 5.1. Variance Loss and Image Blurriness

When comparing input data to generated data for the generic Autoencoders and the VAE, there is a variance loss [54]. This was empirically measured in [54]. This phenomena is possibly due to averaging.

When being used to generate new images, VAEs tend to be more blurry compared to other generative models. Variance loss is a main cause of this [54]. In [55], they find that the maximum likelihood approach is not always the cause of blurriness, it is choice of the inference distribution. They use a sequential VAE model. Choosing flexible inference models or flexible generative models in the architecture also helps to reduce this problem [27].

The VAE-GAN reduces image blurriness by replacing the reconstruction loss term with a discriminator [56]. The multi stage VAE [57], deep residual VAE, and Hierarchical VAEs such as VAE’s with inverse autoregressive flows (IAF-VAE) [58] and Noveau VAE (NVAE) [59] also improve image generation quality. PixelVAE [60], 2-Stage VAE [61], and VQ-VAE are also very effective in generating good quality images.

### 5.2. Disentanglement

How successful machine learning methods are depends on data representation. In [62], they hypothesize that the reason behind this dependence on data representation is that multiple explanatory factors of variations of the data are entangled and hidden by the representation. Representation learning can be defined as learning data representations that makes extracting useful information easier for input into predictors [62]. Three important goals of a good representation include being distributed, invariant, and having disentangled the factors of variation. Disentanglement and disentangled representations do not have agreed upon formal definitions. An intuitive definition that is commonly used is “*a disentangled representation should separate the distinct, informative factors of variations in the data*” [63].

The vanilla VAE fails to learn disentangled representations. INFOVAE [64], β-VAE [65], β-TCVAE [66], AnnealedVAE [67], DIP-VAE I/II [68], and FactorVAE [68] are VAE variants that attempt to obtain a disentangled representation, and many of them are the state of the art for disentanglement. However, according to a large-scale empirical study by Google AI, these state-of-the-art VAE models do not really learn disentangled representations in an unsupervised manner [63]; the choice of the model is not as important as the random seeds and hyperparameters; these hyperparameters do not transfer across data sets.

### 5.3. The Balancing Issue

In the VAE loss function for context of images, the KL Divergence regularizes the latent space, while the reconstruction loss affects the quality of the image [69]. There is a tension between these two effects. If we emphasize the reconstruction loss, the reconstruction is more powerful, but the latent space shape is affected, so the capabilities of the VAE to generate new examples are negatively affected. If we emphasize the regularizing term, the disentangling becomes better and the latent space is smoother and normalized. However, it also results in the images being more blurry.

The 2-Stage VAE uses a balancing factor that it learns during training to balance these effects. In [69], they use a deterministic variable for the decoder variance to balance these factors.

### 5.4. Variational Pruning and Posterior Collapse

Generally, in variational inference, there is a problem called variational pruning. If we rewrite ELBO as the following:

(58) $$L\left(\theta ,\phi ;x\right)=\log\left(p_{\theta }\left(x\right)\right)-D_{KL}\left(q_{\phi }\left(z|x\right)∥p_{\theta }\left(z|x\right)\right)$$

The $D_{KL}\left(q_{\phi }\left(z|x\right)∥p_{\theta }\left(z|x\right)\right)$ term is known as the variational gap. When we maximize ELBO, we either decrease the variational gap or increase the log evidence. Maximum likelihood training increases the log evidence. To decrease the variational gap, there are two options. The first is to update ϕ to make the variational posterior closer to the real posterior. The second way is to update θ so that the real posterior is closer to the variational posterior; this can lead to a decrease in how well the model can fit the data. This effect can be mitigated by using a more expressive posterior.

One possible consequence of this is called variational pruning; this is when latent variables are not used for the model, and the posterior becomes the same as the prior. In variational autoencoders, this is called posterior collapse. Some researchers speculate that the KL Divergence term $D_{KL}\left(q_{\phi }\left(z|x\right)∥p\left(z\right)\right)$ in the ELBO is a cause of this phenomena. Thus, this has led to a focus on reducing the effect of this KL term. The decoder becoming powerful is another cause. Lucas [70] investigated posterior collapse; initially, they investigated it for a linear VAE, and then extended their results for nonlinear VAEs. They find that, in cases where the decoder is not powerful, posterior collapse can still happen. They also formally define posterior collapse and how to measure it.

The δ-VAE [71], Variational Mixture of Posteriors prior VAE (VampPrior VAE) [72], 2-Stage VAE [61], epitomic VAE (eVAE) [73], and VQ-VAE [74] are some models that attempt to deal with preventing posterior collapse.

### 5.5. Origin Gravity Effect

The origin gravity effect is an effect in low dimensions. Since the prior is a multivariate standard normal distribution, the probabilities are centered around the origin. This pushes the points in the latent space towards the origin. Thus, even when the data are spread around multiple clusters, the Gaussian prior tends to push the clusters centers of the latent space toward the origin. Ideally, the latent space should have separate clusters and the prior should not push the mean toward the origin. We can exploit this clustering structure by using GMM based models, such as VADE and GMM-VAE [75,76].

### 5.6. Hidden Score Function

The pathwise gradient has a hidden score function that can lead to high variance; this is discussed more in depth in Section 6.1.

### 5.7. Curse of Dimensionality

Since the Gaussian prior has a $L_{2}$ norm, it suffers from the curse of dimensionality [77]. The mass of the Gaussian distribution is no longer concentrated around the mean when we go to higher dimensions. Instead of a bell curve, a higher dimensional Gaussian resembles a uniform distribution on a surface of a hypersphere; most of the mass is on the shell of the hypersphere. This can cause inefficiencies when sampling in high dimensions [78]. The random walk Metropolis algorithm tends to perform poorly when sampling in high dimensions; the Hamiltonian Monte Carlo tends to perform better.

### 5.8. GANs vs. VAEs

VAEs and GANs use generative models to generate new data. GANs tend to be better at generating images that are perceived by humans to be good quality; however, they do not model the density very well with respect to the likelihood criterion [27]. VAEs are the opposite; they tend to have blurry images but model the density very well with respect to the likelihood criterion. The VAE is more stable to train than the GAN.

## 6. Variations of the VAE

There are many ways to extend the VAE models. You can change the prior, the posterior/variational posterior, regularize the posterior, and change the architecture. Changing the architecture includes changing the layers to RNNs/LSTMs/CNN layers, and use other Divergence measures instead of KL Divergence. Many of these variations will often include convolutional layers, even if not explicitly stated. In this section, we will refer to the original VAE as the vanilla VAE.

### 6.1. VAE Using an STL Estimator

For the VAE, we can decompose the gradient of the lower bound w.r.t ϕ as follows [79]:

$$\begin{array}{cc} {\hat{\nabla }}_{\mathrm{TD}}\left(\epsilon ,\phi \right) & =\nabla_{\phi }\left[\log\left(p_{\theta }\left(x|z\right)\right)+\log\left(p_{\theta }\left(z\right)\right)-\log\left(q_{\phi }\left(z|x\right)\right)\right]\\ & =\nabla_{\phi }\left[\log\left(p_{\theta }\left(z|x\right)\right)+\log\left(p_{\theta }\left(x\right)\right)-\log\left(q_{\phi }\left(z|x\right)\right)\right]\\ & =\underset{\mathrm{path}\;\mathrm{derivative}\;}{\underset{︸}{\nabla_{z}\left[\log\left(p_{\theta }\left(z|x\right)\right)-\log\left(q_{\phi }\left(z|x\right)\right)\right]\nabla_{\phi }g\left(\epsilon ,\phi ,x\right)}}-\underset{\mathrm{score}\;\mathrm{function}\;}{\underset{︸}{\nabla_{\phi }\log\left(q_{\phi }\left(z|x\right)\right)}} \end{array}$$

The score function term can lead to a higher variance than necessary. One way to address this is to drop the score function term, leading to the following term being used instead of the gradient:

(59) $$\nabla_{z}\left[\log\left(p_{\theta }\left(z|x\right)\right)-\log\left(q_{\phi }\left(z|x\right)\right)\right]\nabla_{\phi }g\left(\epsilon ,\phi ,x\right)$$

This does not affect the bias of the estimator because the expectation of the score function is 0. In some cases, dropping it can actually increase the variance if the score function is correlated with the other terms.

We call it the STL estimator because the paper that invented this new estimator was titled “Sticking the Landing: Simple, Lower-Variance Gradient Estimators for Variational Inference”.

### 6.2. ρ-VAE

Instead of an isotropic Gaussian approximate posterior, we use an AR(1) Gaussian distribution [80], so this is a posterior variant. Note by autoregressive Gaussian that we are referring to a traditional autoregressive model. The covariance matrix of the AR(1) process is given by

(60) $$C_{\left(\rho ,s\right)}=s\left[\begin{array}{cccccc} 1 & \rho & \rho^{2} & \rho^{3} & \cdots & \rho^{d-1}\\ \rho & 1 & \rho & \rho^{2} & \cdots & \rho^{d-2}\\ \rho^{2} & \rho & 1 & \rho & \cdots & \rho^{d-3}\\ \rho^{3} & \rho^{2} & \rho & 1 & \cdots & \rho^{d-4}\\ \vdots & \; & & \ddots & \ddots & \vdots \\ \rho^{d-1} & \cdots & \rho^{3} & \rho^{2} & \rho & 1 \end{array}\right]$$

The ρ parameter is a scalar parameter controlling the correlation, so it is between −1 and 1; s > 0 is a scalar scaling parameter. The subscript (ρ,s) denotes that that the covariance matrix is dependent on ρ and s.

The vanilla VAE encoder outputs μ and $\sigma^{2}$ (or log($\sigma^{2}$)); the encoder in the ρ-VAE outputs μ, ρ, and s. The determinant for this matrix is

(61) $$\det\left(C_{\left(\rho ,s\right)}\right)=s^{d}{\left(1-\rho^{2}\right)}^{d-1}$$

The regularization term in the loss function can be formulated as:

$$D_{KL}\left(N\left(\mu^{\left(i\right)},C_{\left(\rho ,s\right)}\right)∥N\left(0,I_{d}\right)\right)=\frac{1}{2}\left[{∥\mu^{\left(i\right)}∥}_{2}^{2}+d\left(s-1-\log\left(s\right)\right)-\left(d-1\right)\log\left(1-\rho^{2}\right)\right]$$

We can take the Cholesky decomposition of the covariance matrix; from there, we get the following lower triangular matrix:

$${\tilde{C}}_{\left(\rho ,s\right)}=\frac{1}{\sqrt{s}}\left[\begin{array}{cccccc} 1 & 0 & 0 & 0 & 0 & 0\\ \rho & \sqrt{1-\rho 2} & 0 & 0 & \cdots & 0\\ \rho^{2} & \rho \sqrt{1-\rho 2} & \sqrt{1-\rho 2} & 0 & \cdots & 0\\ \rho^{3} & \rho^{2}\sqrt{1-\rho 2} & \rho \sqrt{1-\rho 2} & \sqrt{1-\rho 2} & \cdots & 0\\ \vdots & & & \ddots & \ddots & \vdots \\ \rho^{d} & \cdots & \rho^{3}\sqrt{1-\rho 2} & \rho^{2}\sqrt{1-\rho 2} & \rho \sqrt{1-\rho 2} & \sqrt{1-\rho 2} \end{array}\right]$$

$z^{\left(i\right)}=\mu^{\left(i\right)}+{\tilde{C}}_{\left(\rho ,s\right)}^{\left(i\right)}\epsilon$, can be used to generate the latent codes. $z^{\left(i\right)}$ is d-dimensional latent code associated with the *i*th input; ϵ is a d-dimensional vector sampled from a multivariate standard normal distribution, and ${\tilde{C}}_{\left(\rho ,s\right)}^{\left(i\right)}$ is a d x d lower triangular matrix for the ith input. Variations of the ρ-VAE include the ρ−β-VAE and the INFO-β-VAE.

### 6.3. Importance Weighted Autoencoder (IWAE)

#### 6.3.1. Importance Sampling

Importance sampling is a Monte Carlo variance reduction method [81], where you have the following integral to estimate

$$A=\int_{\mathrm{range}(x)}g\left(x\right)f\left(x\right)dx=E_{f}\left[g\left(x\right)\right]$$

where $\mathrm{range}\left(x\right)\subseteq R^{d}$ is bounded $,g:\mathrm{range}\left(x\right)\to R^{d}$ is bounded and integrable; *f*: PDF of a random variable x∈range(x);f≥0 on range(x),f=0 outside of range(x), and ∫f(x)dx=1. We choose probability distribution function γ on range(x);γ≠0 on range(x); this γ is called the importance function.

$\int_{\mathrm{range}(x)}g\left(x\right)f\left(x\right)dx\to \int_{\mathrm{range}(x)}\frac{g(x)f(x)}{\gamma (x)}\gamma \left(x\right)dx=E_{\gamma }\left[\frac{g(y)f(y)}{\gamma (y)}\right]=J$, where y∼γ

The importance sampling Monte Carlo estimator becomes

(62) $$\hat{J}=J_{N}=\frac{1}{N}\sum_{k=1}^{N}\frac{g\left(y_{k}\right)f\left(y_{k}\right)}{\gamma \left(y_{k}\right)}$$

The algorithm is as follows:

(1) Generate i.i.d sequence $\{y_{1},\cdots ,y_{N}\}∼\gamma$.
(2) Plug into Equation (62).

This is an unbiased estimator.

#### 6.3.2. Importance Sampling for a Latent Variable Model

If we are trying to train a latent variable model to perform inference, with random vectors z and x, we can use importance sampling in training the likelihood. If our *f* is our prior distribution $p_{z}\left(z\right)$, and g is the log conditional likelihood $p_{\theta }\left(x^{\left(i\right)}|z\right)$, the expected value we are estimating is

$$E_{\gamma }\left[\frac{p\left(x|y\right)p_{z}\left(y\right)}{\gamma (y)}\right]$$

Then, the importance sampling estimator becomes

$$J_{N}=\frac{1}{L}\sum_{l=1}^{L}\frac{g\left(y^{\left(l\right)}\right)p_{z}\left(y^{\left(l\right)}\right)}{\gamma \left(y^{\left(l\right)}\right)},y^{\left(l\right)}∼\gamma^{\left(l\right)}$$

We would use importance sampling here if our $p_{z}\left(z\right)$ was difficult to sample from, or was not informative. When training the likelihood given by $\sum_{i}\log\left(\sum_{z}p_{z}\left(z\right)p_{\theta }\left(x^{\left(i\right)}|z\right)\right)$,

$\sum_{k}p_{z}\left(z\right)p_{\theta }\left(x^{\left(i\right)}|z\right)$ can be estimated with $J_{N}$, so

$$\sum_{i}\log\left(\sum_{l}p_{z}\left(z\right)p_{\theta }\left(x^{\left(i\right)}|z\right)\right)\approx \sum_{i}\log\left(\frac{1}{L}\sum_{l=1}^{L}\frac{p_{\theta }\left(y^{\left(i,l\right)}\right)}{\gamma \left(y^{\left(i,l\right)}\right)}p_{\theta }\left(x^{\left(i\right)}\left|y^{\left(i,l\right)}\right)\right)\right),y^{\left(i,l\right)}∼\gamma \left(y^{\left(i,l\right)}\right)$$

We choose our importance function to be the variational posterior, $q_{\phi }\left(z|x\right)$.

(63) $$\sum_{i}\log\left(\frac{1}{L}\sum_{l=1}^{L}\frac{p_{z}\left(z^{\left(i,l\right)}\right)}{q\left(z^{\left(i,l\right)}|x\right)}p_{\theta }\left(x^{\left(i\right)}|z^{\left(i,l\right)}\right)\right),z^{\left(i,l\right)}∼q\left(z^{\left(i,l\right)}\right)$$

The IWAE [82] is a variation that uses importance sampling for weighted estimates of the log probability. There are two important terms:

Term 1: $\;\sum_{i}\log\left(\frac{1}{L}\sum_{l=1}^{L}\frac{p_{z}\left(z^{\left(i,l\right)}\right)}{q\left(z^{\left(i,l\right)}\right)}p_{\theta }\left(x^{\left(i\right)}|z^{\left(i,l\right)}\right)\right)$ with $z^{\left(i,l\right)}∼q\left(z^{\left(i,l\right)}\right)$

Term 2: $\;\min_{\phi }\sum_{i}D_{KL}\left(q_{\phi }\left(z|x^{\left(i\right)}\right)∥p_{\theta }\left(z|x^{\left(i\right)}\right)\right)$

We want to maximize Term 1–Term 2. Thus, we end up with

(64) $$\begin{array}{ccc} L_{IWAE}=E_{z^{\left(i,1\right)},z^{\left(i,2\right)},z^{\left(i,3\right)}\cdot \ldots ,z^{\left(i,l\right)}∼q\left(z|x\right)}\left[\log\left(\frac{1}{L}\sum_{l=1}^{L}\frac{p_{\theta }\left(x,z^{\left(i,L\right)}\right)}{q(z^{\left(i,l\right)}|x^{\left(i\right)})}\right)\right] & & \\ \leq \log\left(p_{\theta }\left(x^{\left(i\right)}\right)\right) \end{array}$$

We can do a form of the ELBO by taking L samples of $q_{\phi }\left(z|x\right)$.

$$L_{\mathrm{VAE}}=E_{z^{\left(i,1\right)},z^{\left(i,2\right)},z^{\left(i,3\right)},\ldots ,z^{\left(i,L\right)}∼q\left(z|x\right)}\left[\frac{1}{L}\sum_{l=1}^{L}\log\left(\frac{p_{\theta }\left(x^{\left(i\right)},z^{\left(i,l\right)}\right)}{q(z^{\left(i,l\right)}|x^{\left(i\right)})}\right)\right]\leq \log\left(p_{\theta }\left(x^{\left(i\right)}\right)\right)$$

Using Jensen’s Inequality, we can see that

(65) $$L_{VAE}\leq L_{IWAE}\leq \log\left(p_{\theta }\left(x^{\left(i\right)}\right)\right)$$

The loss for the IWAE forms a tighter bound than the VAE, and, as you increase K, the bound becomes tighter. In the IWAE lower bound, the gradient weighs the datapoint by relative importance. In the VAE lower bound, the weights are equally weighted.

#### 6.3.3. IWAE Variance Reductions

The gradient estimator of the IWAE can still have higher variance than desirable [79,83], due to a hidden score function. To eliminate this problem, you can drop the hidden score function, leading to IWAE-STL [79]. You can also use the reparametrization trick on the hidden score function [84]. This new estimator is called the doubly reparametrized gradient estimator (DReG). This leads to the IWAE-DReG.

### 6.4. Mixture-of-Experts Multimodal VAE (MMVAE)

The Multimodal VAE (MVAE) [85] and MMVAE model [86] address generative modeling of data across multiple modalities. In this context, examples of multimodal data include images with captions and video data with accompanying audio.

We have M modalities, denoted by m=1,…,M of the form

(66) $$p_{\Theta }\left(z,x_{1:M}\right)=p\left(z\right)\prod_{m=1}^{M}p_{\theta_{m}}\left(x_{m}|z\right)$$

where $p_{\theta_{m}}\left(x_{m}|z\right)$ are likelihoods; it is parameterized by a decoder. This decoder has parameters $\Theta =\left\{\theta_{1},\ldots ,\theta_{M}\right\}$.

The true joint posterior is denoted as $p_{\Theta }\left(z|x_{1:M}\right)$, and the variational joint posterior $q_{\Phi }\left(z|x_{1:M}\right)$,

(67) $$q_{\Phi }\left(z|x_{1:M}\right)=\sum_{m=1}^{M}a_{m}q_{\phi_{m}}\left(z|x_{m}\right)$$

where $a_{m}=\frac{1}{M}$ and $q_{\phi_{m}}\left(z|x_{m}\right)$ denotes a unimodal posterior.

We plug this into the $L_{IWAE}$ to get

(68) $$L_{IWAE}^{\mathrm{MoE}}=\frac{1}{M}\sum_{m=1}^{M}E_{z^{1:K}∼q_{\Phi }\left(z|x_{1:M}\right)}\left[\log\left(\sum_{k=1}^{K}\frac{1}{K}\frac{p_{\Theta }\left(z_{m}^{k},x_{1:M}\right)}{q_{\Phi }\left(z_{m}^{k}|x_{1:M}\right)}\right)\right]$$

### 6.5. VR-α Autoencoder and VRmax Autoencoder

We can also derive a variational lower bound for Rényi’s α-Divergence, called variational Rényi (VR) bound [87]. We approximate the exact posterior $p_{\theta }\left(z|x\right)$ for α>0.

$$\min_{q(z)}D_{\alpha }\left(q_{\phi }\left(z|x\right)∥p_{\theta }\left(z|x^{\left(i\right)}\right)\right)$$

$$\max_{q\in Q}L_{\alpha }\left(q;x\right)=\max_{q\in Q}\log\left(p_{\theta }\left(x\right)\right)-D_{\alpha }\left(q_{\phi }\left(z|x\right)∥p_{\theta }\left(z|x\right)\right)$$

when α≠1, it is equivalent to

(69) $$L_{\alpha }\left(q;x\right):=\frac{1}{1-\alpha }\log E_{q}\left[{\left(\frac{p(z,x)}{q(z|x)}\right)}^{1-\alpha }\right]$$

This definition can be extended to α≤0, The VR-α Autoencoder minimizes this VR bound. The VRmax Autoencoder is an autoencoder in the case of the Renyi Divergence where α=−∞.

IWAE can also be seen as the case of the Renyi Divergence when α=0 and L < *∞*; L is the sample size of the Monte Carlo estimator. When α = 1, the VR-α Autoencoder becomes the vanilla VAE.

### 6.6. INFOVAE

The INFOVAE [64], also known as MMD-VAE, is a posterior regularizing variant. This leads to better disentangled representations. However, the INFOVAE still has the blurred images generation problem.

The term $q_{\phi }\left(x|z\right)$ is the posterior to $q_{\phi }\left(z|x\right)$ and $p_{\theta }\left(z|x\right)$ is the posterior to $p_{\theta }\left(x|z\right)$.

The Divergence between *q* and *p*, $D_{KL}\left(q_{\phi }\left(z\right)∥p\left(z\right)\right)$, is multiplied by λ, a scaling parameter. A mutual information between *x* and *z* under *q*, denoted by $I_{q}\left(x;z\right)$, is also added, and scaled by parameter α (this α is different from the α in Renyi Entropy and Divergence). This gives us the following loss function:

(70) $$\begin{array}{ccc} L_{\mathrm{InfoVAE}\;}=-\lambda D_{KL}\left(q_{\phi }\left(z|x\right)∥p\left(z\right)\right)-E_{q(z)}\left[D_{KL}\left(q_{\phi }\left(x|z\right)∥p_{\theta }\left(x|z\right)\right)\right] & & \\ +\alpha I_{q}\left(x;z\right) \end{array}$$

This objective function cannot be optimized directly; an equivalent form is

(71) $$\begin{array}{ccc} L_{\mathrm{InfoVAE}\;}\equiv E_{p_{\theta }\left(x\right)}E_{q_{\phi }\left(z|x\right)}\left[\log(p_{\theta }\left(x|z\right))\right]-\left(1-\alpha \right)E_{p_{\theta }\left(x\right)}D_{KL}\left(q_{\phi }\left(z|x\right)∥p\left(z\right)\right)- & & \\ \left(\alpha +\lambda -1\right)D_{KL}\left(q_{\phi }\left(z\right)∥p\left(z\right)\right) \end{array}$$

One typical architecture configuration for the INFOVAE involves using a DCGAN for both the encoder and the decoder.

### 6.7. β-VAE

The β-VAE [65] is a posterior regularizing variant. We weight the regularizing term by β, so the ELBO is modified to:

(72) $$E_{q_{\phi }\left(z|x\right)}\left[\log\left(p_{\theta }\left(x|z\right)\right)\right]-\beta D_{KL}\left(q_{\phi }\left(z|x\right)∥p_{z}\left(z\right)\right)$$

β is typically greater than 1. The correct choice of β creates more disentangled latent representation. However, the balancing issue comes in to play; there is a trade-off between reconstruction fidelity and the disentanglement of the latent code [67]. In [67,88,89], the β-VAE’s ability to disentangle has been analyzed. In [67], the authors explore disentanglement through the information bottleneck perspective and propose modifications to increase the disentanglement capabilities of the β-VAE.

### 6.8. PixelVAE

The PixelVAE [60] is a VAE based model with a decoder based on the PixelCNN. Since the PixelCNN is a neural autoregressive model, the decoder of the PixelVAE is a neural autoregressive decoder.

The encoder and decoder both have convolutional layers. The convolutional layers use strided convolutions in the encoder for downsampling. The convolutions in the decoder and are transposed for upsampling.

Typically, a VAE decoder models each output dimension independently, so they use factorizable distributions. In the PixelVAE, a conditional PixelCNN is used in the decoder. The decoder is modeled by:

(73) $$p\left(x|z\right)=\prod_{i}p\left(x_{i}|x_{1},\ldots ,x_{i-1},z\right)$$

We model the distribution of *x* as the product of the distributions of each dimension of *x*, denoted by $x_{i}$, conditioned by *z* and all of previous dimensions. The variable *z* is the latent variable.

**Figure 7:**
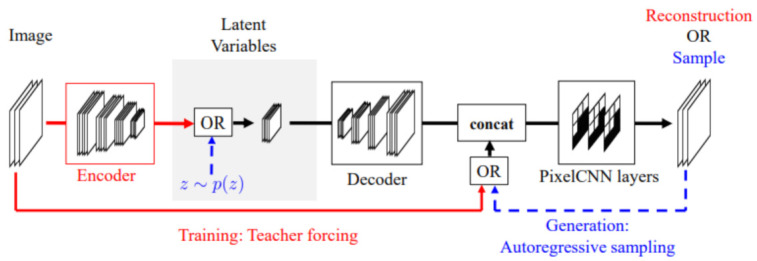
PixelVAE architecture. Image taken from [60].

The PixelCNN is great at capturing details but does not have a latent code. The VAE is great at learning latent representations and capturing a global structure; it is not great at capturing details. The PixelVAE has the positives of both models; it has a latent representation, and is great at capturing global structure and small details. It can also have latent codes that are more compressed than the vanilla VAE. Figure 7 shows the architecture of the PixelVAE.

The performance of VAEs can be improved by creating a hierarchy of random latent variables through stacking VAEs. This idea can also be applied to the PixelVAE.

The PixelVAE++ algorithm uses PixelCNN++ instead of PixelCNN in the decoder [90]. It also uses a discrete latent variables with a Restricted Boltzmann Machine prior.

### 6.9. HyperSpherical VAE/S-VAE

The vanilla VAE often fails to model data whose latent structure is hyperspherical. The soap bubble effect and the gravity origin effect are also a problem with Gaussian priors in the VAE. The HyperSpherical VAE [77] attempts to deal with these problems.

The von Mises Fisher (vmF) distribution is parameterized by $\mu \in R^{m}$ and $\kappa \in R_{\geq 0};\mu$ is the mean direction, and κ is the concentration around the mean. The PDF of a vmF distribution for random vector $z\in R^{m}$:

(74) $$\begin{array}{cc} q(z;\mu ,\kappa ) & =C_{m}\left(\kappa \right)\exp\left(\kappa \mu^{T}z\right)\\ C_{m}\left(\kappa \right) & =\frac{\kappa^{m/2-1}}{{\left(2\pi \right)}^{m/2}I_{m/2-1}\left(\kappa \right)} \end{array}$$

$I_{v}$ represents a modified Bessel function of the first kind at order v.

The hyperspherical VAE uses the vMF as a the variational posterior. The primary advantage of this is the ability to use a uniform distribution as the prior. The KL Divergence term $D_{KL}\left(\mathrm{vMF}\left(\mu ,\kappa \right)∥U\left(S^{m-1}\right)\right)$ to be optimized is:

(75) $$\kappa \frac{I_{m/2}\left(k\right)}{I_{m/2-1}\left(k\right)}+\log\left(C_{m}\left(\kappa \right)\right)-\log{\left(\frac{2\left(\pi^{m/2}\right)}{\Gamma (m/2)}\right)}^{-1}$$

The KL term does not depend on μ, this parameter is only optimized in the reconstruction term. The gradient with respect to the κ is

(76) $$\begin{array}{c} \nabla_{\kappa }D_{KL}\left(\mathrm{vMF}\left(\mu ,\kappa \right)∥U\left(S^{m-1}\right)\right)=\frac{1}{2}k\left(\frac{I_{m/2+1}\left(k\right)}{I_{m/2-1}\left(k\right)}\right.\\ \left.-\frac{I_{m/2}\left(k\right)\left(I_{m/2-2}\left(k\right)+I_{m/2}\left(k\right)\right)}{I_{m/2-1}{\left(k\right)}^{2}}+1\right) \end{array}$$

The sampling procedure for the vmF can be found in [91]. The N-Transformation reparameterization trick can be used to extend the reparameterization trick to more distributions [92]; it is used to reparameterize vmF sampling.

### 6.10. δ-VAE

$D_{KL}\left(q_{\phi }\left(z|x\right)∥p\left(z\right)\right)$ is also called the rate term. The δ-VAE [71] attempts to prevent posterior inference by preventing the rate term from going to 0 by using a lower bound. They address the posterior collapse problem with structural constraints so that the KL Divergence between the posterior and prior is lower bounded by design. This can be achieved by choosing families of distributions for $p_{\theta }\left(z\right)$ and $q_{\phi }\left(z|x\right)$ such that

(77) $$\min_{\theta ,\phi }D_{KL}\left(q_{\phi }\left(z|x\right)∥p_{\theta }\left(z\right)\right)\geq \delta$$

The committed rate of the model is denoted by δ. One way to do so is to select from a family of Gaussian distributions with variances that are fixed but different.

### 6.11. Conditional Variational Autoencoder

The conditional VAE [93] is a type of deep conditional generative model (CGM). In a deep CGM, there are three types of variables: x denotes the input variables, y denotes the output variables, and z denotes the latent variables. The approximate posterior is is $q_{\phi }\left(z|x,y\right)$. The conditional prior is $p_{\theta }\left(z|x\right)$, and the conditional likelihood is $p_{\theta }\left(y|x,z\right).$

After x is observed, z is sampled from $p_{\theta }\left(z|x\right)$. Then, y is generated from $p_{\theta }\left(y|x,z\right).$ The variational lower bound of the deep CGM is

(78) $$\log\left(p_{\theta }\left(y|x\right)\right)\geq -D_{KL}\left(q_{\phi }\left(z|x,y\right)∥p_{\theta }\left(z|x\right)\right)+E_{q_{\phi }\left(z|x,y\right)}\left[\log(p_{\theta }\left(y|x,z\right))\right]$$

For the CVAE, where L is the number of samples, $z^{\left(l\right)}=g_{\phi }\left(x,y,\epsilon^{\left(l\right)}\right),\epsilon^{\left(l\right)}∼N\left(0,I\right)$, the lower bound estimator is

(79) $${\tilde{L}}_{\mathrm{CVAE}}\left(x,y;\theta ,\phi \right)=-D_{KL}\left(q_{\phi }\left(z|x,y\right)∥p_{\theta }\left(z|x\right)\right)+\frac{1}{L}\sum_{l=1}^{L}\log\left(p_{\theta }\left(y|x,z^{\left(l\right)}\right)\right)$$

The encoder is $q_{\phi }\left(z|x,y\right)$, the conditional prior is $p_{\theta }\left(z|x\right)$, and the decoder is $p_{\theta }\left(y|x,z\right).$

### 6.12. VAE-GAN

The VAE-GAN architecture [56] is influenced by both the VAE and the GAN; the decoder is also the generator, and the reconstruction loss term is replaced by a discriminator.

**Figure 8:**
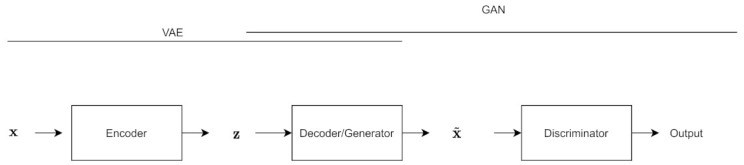
Diagram of the VAE-GAN architecture. x is the input to the encoder, which outputs latent variable z, which goes into the decoder/generator. This is fed into the discriminator. The decoder/generator is part of both the VAE and GAN.

As shown in Figure 8, there you have the same VAE structure, but the sample coming out of the VAE is fed into a discriminator, along with the original training data.

In this model, z is the output of the encoder, denoted z∼Enc(x)=q(z|x), and $\tilde{x}$ is the output of the decoder, denoted by $\tilde{x}∼\mathrm{Dec}\left(z\right)=p\left(x|z\right).$ ${\mathrm{Dis}}_{l}\left(x\right)$ denotes the representation of the *l* th layer of the discriminator.

The likelihood of the lth layer of the discriminator can be given by

$$p\left({\mathrm{Dis}}_{l}\left(x\right)|z\right)=N\left({\mathrm{Dis}}_{l}\left(x\right);{\mathrm{Dis}}_{l}\left(\tilde{x}\right),I\right)$$

It is a Gaussian distribution parametrized with mean ${\mathrm{Dis}}_{l}\left(\tilde{x}\right)$ and the identity matrix I as its covariance. The likelihood loss for ${\mathrm{Dis}}_{l}\left(x\right)$ can be calculated as

$$L_{\mathrm{llike}\;}^{\mathrm{Dis}{\;}_{l}}=-E_{q(z|x)}\left[\log(p\left({\mathrm{Dis}}_{l}\left(x\right)|z\right))\right]$$

The loss of the GAN is typically given by $L_{\mathrm{GAN}}=\log\left(\mathrm{Dis}\left(x\right)\right)+\log\left(1-\mathrm{Dis}\left(\mathrm{Gen}\left(z\right)\right)\right)$. Since the generator and decoder are the same for the VAE-GAN, it can be rewritten as

$$L_{\mathrm{GAN}}=\log\left(\mathrm{Dis}\left(x\right)\right)+\log\left(1-\mathrm{Dis}\left(\mathrm{Dec}\left(z\right)\right)\right)$$

The overall loss used for training the VAE-GAN is

(80) $$L=D_{KL}\left(q\left(z|x\right)∥p\left(z\right)\right)+L_{\mathrm{llike}\;}^{\mathrm{Dis}{\;}_{l}}+L_{\mathrm{GAN}\;}$$

There are multiple practical considerations regarding training the VAE-GAN. The first consideration is to limit propagation of error signals to only certain networks. $\theta_{\mathrm{Enc}\;},\theta_{\mathrm{Dec}\;},\theta_{\mathrm{Dis}\;}$ denote the parameters of each network.

The second consideration is to weigh the error signals that the decoder receives. The decoder receives these signals from both $L_{\mathrm{llike}\;}^{\mathrm{Dis}{\;}_{l}}$ and $L_{\mathrm{GAN}}$,

The parameter η is used as a weighting factor, and the update of the decoders parameters looks like:

$$\theta_{\mathrm{Dec}}\leftarrow -\nabla_{\theta_{\mathrm{Dec}}}\left(\eta L_{\mathrm{llike}\;}^{{\mathrm{Dis}}_{l}}-L_{\mathrm{GAN}}\right)$$

Empirically, the VAE-GAN performs better if the discriminator input includes samples from both p(z) and q(z|x). Therefore, the GAN loss can be rewritten as:

$$\begin{array}{cc} L_{\mathrm{GAN}}= & \log(\mathrm{Dis}(x))+\log(1-\mathrm{Dis}(\mathrm{Dec}(z)))\\ & +\log(1-\mathrm{Dis}(\mathrm{Dec}(\mathrm{Enc}(x)))) \end{array}$$

**Figure 9:**
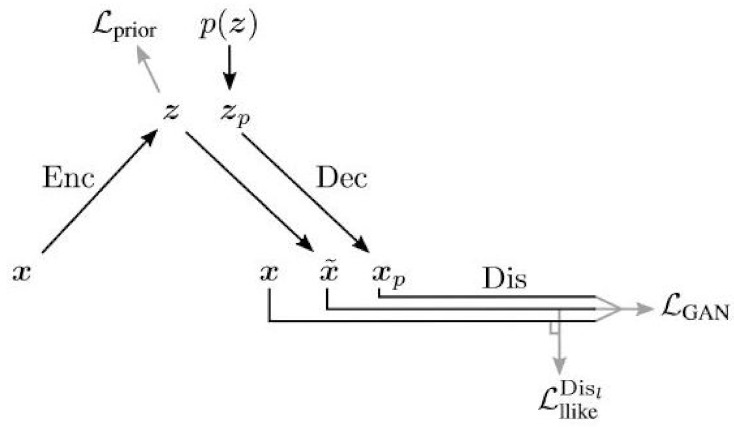
Diagram of the VAE-GAN training, taken from [56].

Algorithm 3 shows the VAE-GAN training algorithm given practical considerations, and Figure 9 shows the architecture given these modifications.
**Algorithm 3:** VAE-GAN training$\theta_{\mathrm{Enc}\;},\theta_{\mathrm{Dec}\;},\theta_{\mathrm{Dis}\;}\leftarrow \;\mathrm{initialize}\;\mathrm{network}\;\mathrm{parameters}\;\mathrm{for}\;\mathrm{encoder},\;\mathrm{decoder},\;\mathrm{and}\;\mathrm{discriminator}\;$ networks**for**do#oftrainingiterations:    $X^{\left(M\right)}\leftarrow \;\mathrm{random}\;\mathrm{mini}-\mathrm{batch}\;$    $Z^{\left(M\right)}\leftarrow \mathrm{Enc}\left(X^{\left(M\right)}\right)$    $L_{\mathrm{prior}\;}\leftarrow D_{KL}(q\left(Z^{\left(M\right)}|X^{\left(M\right)}\right)∥p\left(Z^{\left(M\right)}\right)$    ${\tilde{X}}^{\left(M\right)}\leftarrow \mathrm{Dec}\left(Z^{\left(M\right)}\right)$    $L_{\mathrm{Llike}\;}^{\mathrm{Disl}\;}\leftarrow -E_{q(Z^{\left(M\right)}|X^{\left(M\right)})}\left[p\left({\mathrm{Dis}}_{l}\left(X^{\left(M\right)}\right)|Z^{\left(M\right)}\right)\right]$    $Z_{p}^{\left(M\right)}\leftarrow \;\mathrm{samples}\;\mathrm{from}\;\mathrm{prior}\;N\left(0,I\right)$    $X_{p}^{\left(M\right)}\leftarrow \mathrm{Dec}\left(Z_{p}^{\left(M\right)}\right)$    $L_{\mathrm{GAN}}\leftarrow \log\left(\mathrm{Dis}\left(X^{\left(M\right)}\right)\right)+\log\left(1-\mathrm{Dis}\left({\tilde{X}}^{\left(M\right)}\right)\right)$    $\;+\log\left(1-\mathrm{Dis}\left(X_{p}^{\left(M\right)}\right)\right)$    Updatethenetworkparameterswiththeirstochasticgradients:    $\theta_{\mathrm{Enc}\;}\overset{+}{\leftarrow }-\nabla_{\theta_{\mathrm{Enc}\;}}\left(L_{\mathrm{prior}\;}+L_{\mathrm{llike}\;}^{\mathrm{Dis}{\;}_{l}}\right)$    $\theta_{\mathrm{Dec}}\overset{+}{\leftarrow }-\nabla_{\theta_{\mathrm{Dec}\;}}\left(\eta L_{\mathrm{llike}\;}^{\mathrm{Dis}{\;}_{l}}-L_{\mathrm{GAN}}\right)$    $\theta_{\mathrm{Dis}}\overset{+}{\leftarrow }-\nabla_{\theta_{\mathrm{Dis}}}L_{\mathrm{GAN}}$**end for**

Extensions of the VAE-GAN include the Zero-VAE-GAN [94], F-VAEGAN-D2 [95], 3DVAE-GAN [96], and Hierarchical Patch VAE-GAN [97].

### 6.13. Adversarial Autoencoders (AAE)

The adversarial autoencoder is another architecture that takes inspiration from both the VAE and GAN [98]. We denote x as the input and z as the latent code of the autoencoder. Then, p(z) is the prior probability distribution over the latent code, q(z|x) is the probability distribution for the encoder, p(x|z) is the distribution for the decoder, $p_{d}\left(x\right)$ denotes the data generating distribution, and p(x) is the model distribution. The encoder has an aggregated posterior distribution defined as

$$q\left(z\right)=\int_{x}q\left(z|x\right)p_{d}\left(x\right)dx$$

An adversarial network is connected to the latent code. From there, we sample from the aggregated posterior and the prior, and input both into the discriminator. The discriminator tries to distinguish whether or not the z is from the prior, which means it is real, or if it is from the aggregrated variational posterior, which is fake. This matches the prior with the aggregrated variational posterior, which has a regularizing effect on the autoencoder. The encoder can also be considered the generator of the adversarial net because it is generating the latent code. The autoencoder part of the AAE tries to minimize the reconstruction error.

The AE and the adversarial network are trained in two phases. The first phase is the reconstruction phase. This is where the autoencoder is trained on minimizing the reconstruction loss. The second phase is the regularization phase. In this phase, the discriminative network is trained to discriminate between the real samples and the fake ones. Then, the generator (the encoder of the AE) is also trained to fool the discriminator better. Both of these steps use minibatch SGD.

There is a broad choice of functions for the approximate posterior. Some common choices are a deterministic function, a Gaussian probability distribution, or a universal approximator of the posterior.

**Figure 10:**
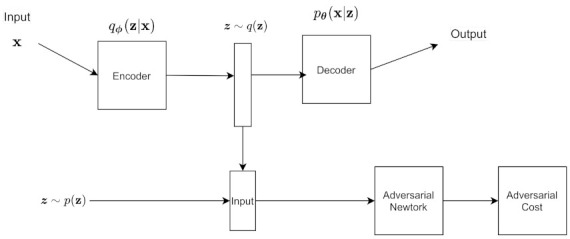
Adversarial Autoencoder architecture. The top part is the autoencoder, while the encoder and the bottom part constitute the GAN.

Figure 10 shows the architecture of the AAE. We can adjust the architecture of the AAE to do supervised learning, semi supervised learning, unsupervised clustering, and dimensionality reduction.

### 6.14. Information-Theoretic Learning Autoencoder

The Information-Theoretic Learning Autoencoder (ITL-AE) [99] is similar to the VAE, with both and encoder and decoder layers. There are two main differences. One is that it does not use the reparameterization trick.

The second difference is that, instead of using the KL Divergence, it uses alternate Divergence measures, like the CS Divergence and the Euclidean Divergence; these Divergences are estimated through kernel density estimation (KDE) [100].

(81) $$\mathrm{cost}=L\left(x,\tilde{x}\right)+\lambda R\left(Enc,p\right)$$

where L is the reconstruction loss, R is the regularization term, typically the Euclidean or CS Divergence. The chosen prior is *p*, Enc is the encoder, and λ controls the magnitude of the regularization.

If we want to estimate the QIP V for p(x) using KDE, it is given by the formula

(82) $${\hat{V}}_{p}=\frac{1}{N^{2}}\sum_{j=1}^{N}\sum_{i=1}^{N}G\left(x_{i}|x_{j},\sigma^{2}\right)$$

where there are N datapoints, and a Gaussian kernel G with kernel bandwidth $\sigma^{2}$. Minimizing the QIP is equivalent to maximizing the quadratic Entropy.

To get the kernel density estimator for the CS-Divergence:

(83) $${\hat{D}}_{CS}\left(q;p\right)=\log\left(\frac{{\hat{V}}_{q}{\hat{V}}_{p}}{{\hat{V}}_{c}^{2}}\right)$$

${\hat{V}}_{q}$ is the QIP estimator for PDF $q\left(x\right),{\hat{V}}_{p}$ is the QIP estimator for PDF $p\left(x\right),{\hat{V}}_{c}$ the cross information potential estimator, given by

(84) $${\hat{V}}_{c}=\frac{1}{N_{q}N_{p}}\sum_{j=1}^{N_{p}}\sum_{k=1}^{N_{q}}G\left(x_{q_{k}}|x_{p_{j}},2\sigma^{2}\right)$$

where $N_{q}$ is the number of observations for distribution q(x), $N_{p}$ is the number of observations for distribution p(x), $G\left(x_{q_{k}}|x_{p_{j}},2\sigma^{2}\right)$ is a Gaussian kernel between points $x_{q_{k}}$ and $x_{p_{j}}$, with a kernel bandwidth $2\sigma^{2}$.

KDE for Euclidean Divergence estimator is given by

(85) $${\hat{D}}_{ED}\left(q;p\right)={\hat{V}}_{q}+{\hat{V}}_{p}-2{\hat{V}}_{c}$$

We can choose *q* as the approximation distribution, and *p* as the prior distribution. When we try to minimize the information potential with respect to *q*, the samples that are generated from *q* would be spread out; when we try to maximize the cross information potential with respect to *q*, the samples from *q* and *p* are closer together. Thus, there is tension between these two effects.

The authors of [99] experimented with three different priors: Laplacian distribution, 2D Swissroll, and a Gaussian distribution, and experimented on MNIST data generation. The Euclidean Divergence did not perform as well as the CS Divergence when the data became high dimensional; high dimensionality also means the batch size has to be larger for the ITL-AE.

### 6.15. Other Important Variations

Important architectures we have not covered include

(1) The VRNN and VRAE: The VRNN [101] is a RNN with a VAE in each layer. There is also the Variational Recurrent Auto-Encoder (VRAE) [102].
(2) VaDe and GMVAE: Both methods use Gaussian Mixture Models; the specific use case is for clustering and generation [75,76].
(3) VQ-VAE: This method combines Vector Quantization (VQ) with the variational autoencoder [74]. Both the posterior and prior are categorical distributions, and the latent code is discrete. An extension of the VQ-VAE is the VQ-VAE2 [103]. These methods are comparable to GANs in terms of image fidelity.
(4) VAE-IAF: This uses inverse autoregressive flow with the VAE [58].
(5) Wasserstein Auto-Encoder (WAE):
  The WAE minimizes a form of the Wasserstein distance between the model PDF and the target PDF [104].
(6) 2-Stage VAE

The 2-Stage VAE [61] addresses multiple problems: image blurriness and the balancing issue. It also can tackle the problem of a mismatch between the aggregrate posterior and expected prior. It trains two different VAEs sequentially. The first VAE learns how to sample from the variational posterior without matching q(z)=p(z). The second VAE attempts to sample from the true q(z) without using p(z).

## 7. Applications

VAEs are typically used for generating data, including images and audio; another common application is for dimensionality reduction. There are many example applications we could have chosen. However, we decided to focus on three: financial, speech source separation, and biosignal applications. The financial applications for VAEs is a new area of research with a huge potential for innovation. Source separation has long been an important problem in the signal processing community, so it is important to survey how the VAE performs in this application. Innovations in biosignal research has a great potential for positive impact for patients with disabilities and disorders; VAEs can help improve the performance of classifiers in biosignal applications through denoising and data augmentation.

### 7.1. Financial Applications

One application is described in [105], where the β -VAE is used to complete volatility surfaces and generate synthetic volatility surfaces for options (in the context of finance). Volatility is the standard deviation of the return on an asset. In finance, options are a contract between two parties that “*gives the holder the right to trade in the future at a previously agreed price but takes away the obligation*” [106]. This is for the simple options; there are more complex options. A volatility surface is a volatility function based on moneyness and time to maturity. For moneyness, delta is used; in the context of finance, delta is the derivative of an option price with respect to the underlying asset.

**Figure 11:**
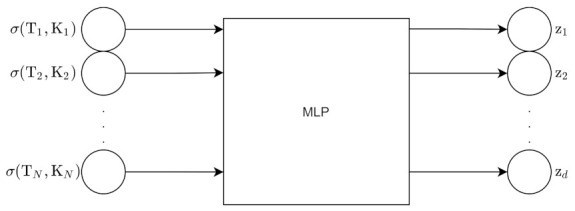
Encoder for grid-based approach.

**Figure 12:**
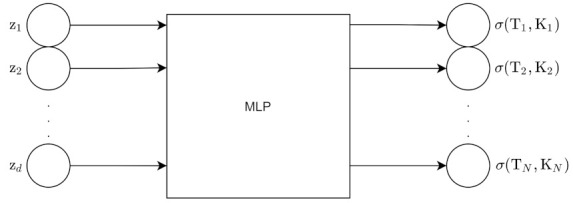
Decoder for grid-based approach.

We sample N points from the volatility surface. There are two types of methods to generate volatility surfaces with the VAE: the grid-based approach and pointwise approach. For the grid-based approach, the input to the encoder is the N grid point surface; this surface is flattened into an N point vector. The encoder outputs z which has d dimensions. The decoder uses z to reconstruct the original grid points. Figure 11 and Figure 12 show the architecture for the encoder and decoder for this approach.

**Figure 13:**
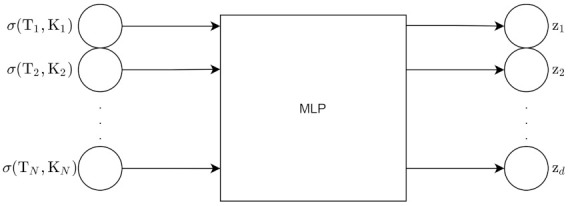
Encoder for pointwise approach.

**Figure 14:**
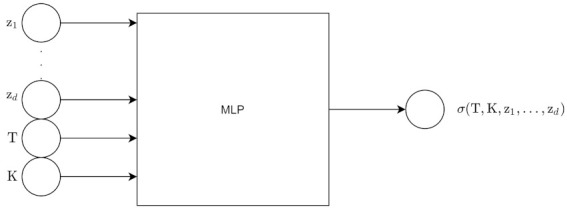
Decoder for pointwise approach.

For the pointwise approach, the input to the encoder is the N grid point surface, which is then flattened into an N point vector. The encoder outputs z, which is d dimensions. The input to the decoder is z along with moneyness K and maturity T. The output of the decoder is 1 point on the volatility surface. We obtain all the points using batch inference. Figure 13 and Figure 14 show the architecture for the encoder and decoder for this approach.

In the experiments, each volatility surface was a 40 points grid, with eight times to maturity and five deltas. Six currency pairs were used in this experiment. Only the pointwise approach was used. For completing surfaces, it was compared with the Heston Model; this algorithm predicts the surface faster than the Heston Model. In some cases, it outperforms the Heston Model. Table 1 shows the results from the paper comparing the Heston Model with a Variational Autoencoder.

The experiments also generated new volatility surfaces. One main use of generating these surfaces is for data augmentation to create more observations for deep learning models (Maxime Bergeron, Private Communications).

In [107], the β-VAE was used in conjunction with continuous time stochastic differential equations models to generate arbitrage-free implied volatility (IV) surfaces. SDE models that were tested included Lévy additive processes and time-varying regime switching models.

**Figure 15:**
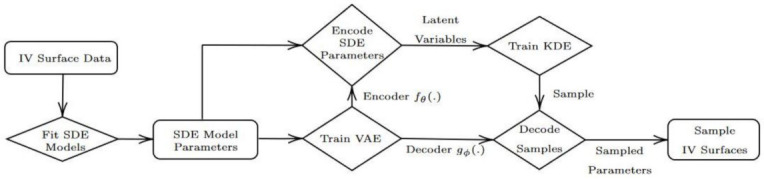
Hybrid model diagram, taken from [107].

The method, shown in the chart in Figure 15, has the following steps:

(1) Use historical market data to fit the arbitrage-free SDE model,
  get collection SDE model parameters.
(2) Train VAE model using on the SDE model parameters.
(3) Sample from the latent space of the VAE model using a KDE approach.
(4) Get a collection of the SDE model parameters by decoding the samples.
(5) Use the SDE model with parameters to get arbitrage-free surfaces.

In [108], LSTM and LightGBM were used to predict the hourly directions of eight banking stocks listed in the BIST 30 Index from 2011 and 2015. The first three years were used in the training set, and the last 2 years were used in the test set. The first experiment used the stock features as the input to the models. The second experiment first put the stock features through a VAE for dimensionality reduction before inputting it into the models. The results found that they performed similarly, though the VAE filtered input uses 16.67% less features. The 3rd experiment involved adding features from other stocks into the first and second experiments, to account for effects from other stocks.

In [109], a deep learning framework was used for multi-step-ahead prediction of the stock closing price. The input features were market open price, market high price, market low price, market close price, and market volume price. This framework used the LSTM-VAE to remove noise, then combined these reconstructed features with original features; these were the input to a stacked LSTM Autoencoder, which outputted a prediction.

In [110], they looked at the index tracking performance of various autoencoder models, including the sparse AE, contractive AE, stacked AE, DAE, and VAE. These were used to find the relationships between stocks and construct tracking portfolios. The results were then compared to conventional methods, and results showed that, in order for the deep learning methods to perform better, there needed to be a higher number of stocks in the tracking portfolio.

### 7.2. Speech Source Separation Applications

Deep learning has been applied to various aspects of speech processing, speech recognition, speech and speaker identification and such applications [111]. In this subsection, we focus on speech source separation applications using variational autoencoders.

**Figure 16:**
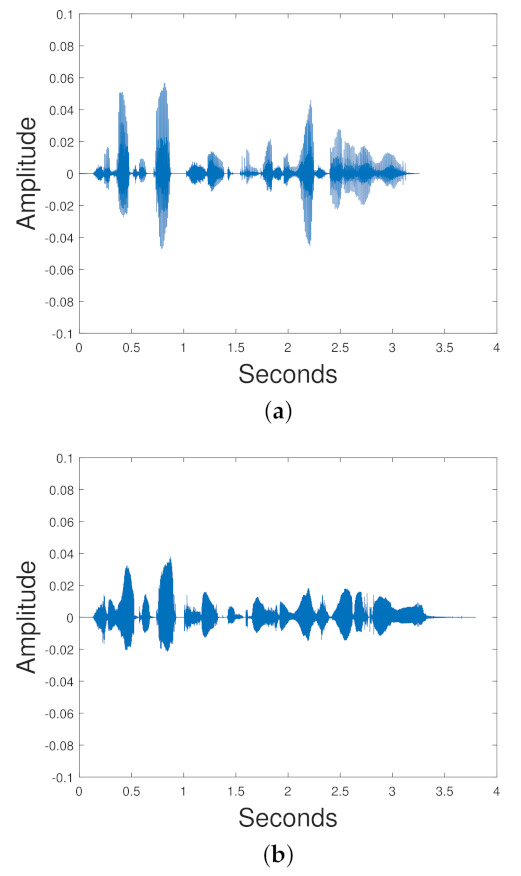

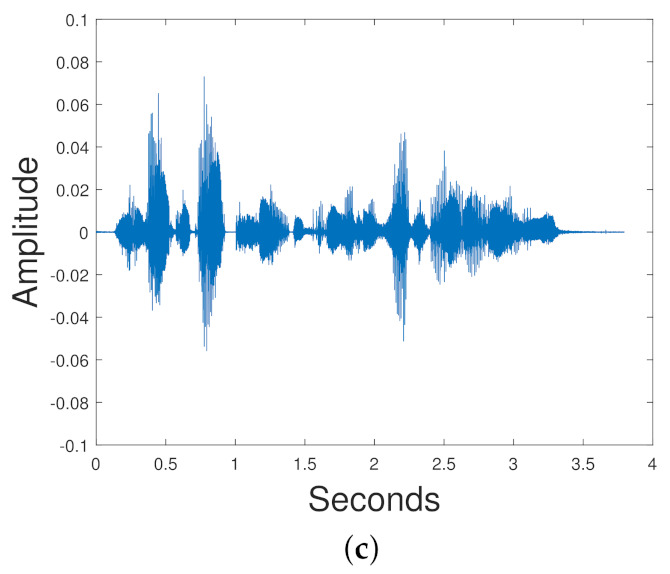
Signal 1 and Signal 2 are mixed together to create the mixed signal. (**a**) Signal 1, (**b**) Signal 2, (**c**) Mixed Signal.

If you have N signals, $s_{i}\left(t\right)$, you can have mixed signal $y\left(t\right)=\sum_{i=1}^{N}\;s_{i}\left(t\right);$ the goal of signal/source separation is to retrieve an estimate of each $s_{i}\left(t\right),\hat{s_{i}}\left(t\right)$. When it is unsupervised, it is known as blind source separation. Figure 16 shows two speech signals, Signal 1 and Signal 2 mixed to create a mixed signal.

Signal to Distortion Ratio (SDR), Signal to Artifact Ratio (SAR), Signal to Interference Ratio (SIR), Signal to Noise Ratio (SNR), and Perceptual Evaluation of Speech Quality (PESQ) are common measures to evaluate speech source separation [112,113]. SDR, SAR, SIR, and SNR are all typically measured in decibels (dB). We will drop the *i* subscript of a signal estimate $\hat{s_{i}}\left(t\right)$ for simplicity in the following formulas. Using the BSSEval toolbox, we can decompose our estimate of a signal as

$$\hat{s}\left(t\right)=s_{\mathrm{target}\;}\left(t\right)+e_{\mathrm{interf}\;}\left(t\right)+e_{\mathrm{noise}\;}\left(t\right)+e_{\mathrm{artif}\;}\left(t\right)$$

$s_{\mathrm{target}\;}\left(t\right)$ is a deformed version of $s_{i}\left(t\right)$, $e_{\mathrm{artif}\;}\left(t\right)$ is an artifact term, $e_{\mathrm{interf}\;}\left(t\right)$ denotes the deformation of the signals due to interference from the unwanted signals, $e_{\mathrm{noise}\;}\left(t\right)$ is a deformation of the perturbating noise.

The SDR is then given by:

(86) $$\mathrm{SDR}=10\log_{10}\frac{{∥s_{\mathrm{dist}\;}∥}^{2}}{{∥e_{\mathrm{interf}\;}+e_{\mathrm{noise}\;}+e_{\mathrm{artif}\;}∥}^{2}}$$

The SIR is given by:

(87) $$\mathrm{SIR}=10\log_{10}\frac{{∥s_{\mathrm{dist}\;}∥}^{2}}{{∥e_{\mathrm{interf}\;}∥}^{2}}$$

the SNR is given by:

(88) $$\mathrm{SNR}=10\log_{10}\frac{{∥s_{\mathrm{dist}\;}+e_{\mathrm{interf}\;}∥}^{2}}{{∥e_{\mathrm{noise}\;}∥}^{2}}$$

and the SAR is:

(89) $$\;\mathrm{SAR}\;=10\log_{10}\frac{{∥s_{\mathrm{dist}\;}+e_{\mathrm{interf}\;}+e_{\mathrm{noise}\;}∥}^{2}}{{∥e_{\mathrm{artif}\;}∥}^{2}}$$

Spectrograms can be used to view a signal’s time-frequency representation. The amplitude and frequency information are represented by color intensity indicating the amplitude of the frequency. A common way of generating spectrograms is to take the Short Time Fourier Transform (STFT) of the signal. Two important parameters for the STFT are the window size and overlap size. The spectrogram is found by taking the square of the magnitude of the STFT especially for deep learning algorithms involving speech. Typically, in practice, the spectrogram is often normalized before being fed into a neural network [111]. Alternative inputs include log spectrograms and mel spectrograms.

In [114], the VAE was compared with NNMF, GAN, Gaussian WGAN, including Autoencoding WGANs (AE WGAN), and Autoencoders trained with maximum likelihood (MLAE) in the task of blind monoaural source separation. This VAE did not use convolutional layers. The TIMIT data set was used [115]. Each training set had a female and male speech signal mixed together. There is a VAE for each speaker; so, for a mixed signal that mixes a female and male speaker, we need two VAEs. The input to the VAE is a normalized spectrogram. The training label is the ground truth spectrogram of the original signal that we are trying to obtain. The signal is reconstructed via the Wiener filtering equation:

(90) $${\hat{x}}_{k}\left(t\right)=\mathrm{iSTFT}\left(\frac{{\hat{S}}_{k}}{{\hat{S}}_{m}+{\hat{S}}_{f}}⊙S_{\mathrm{mixed}\;}⊙e^{i(\;\mathrm{phase}\;)}\right),\;k\in \left\{m,f\right\}$$

**Figure 17:**
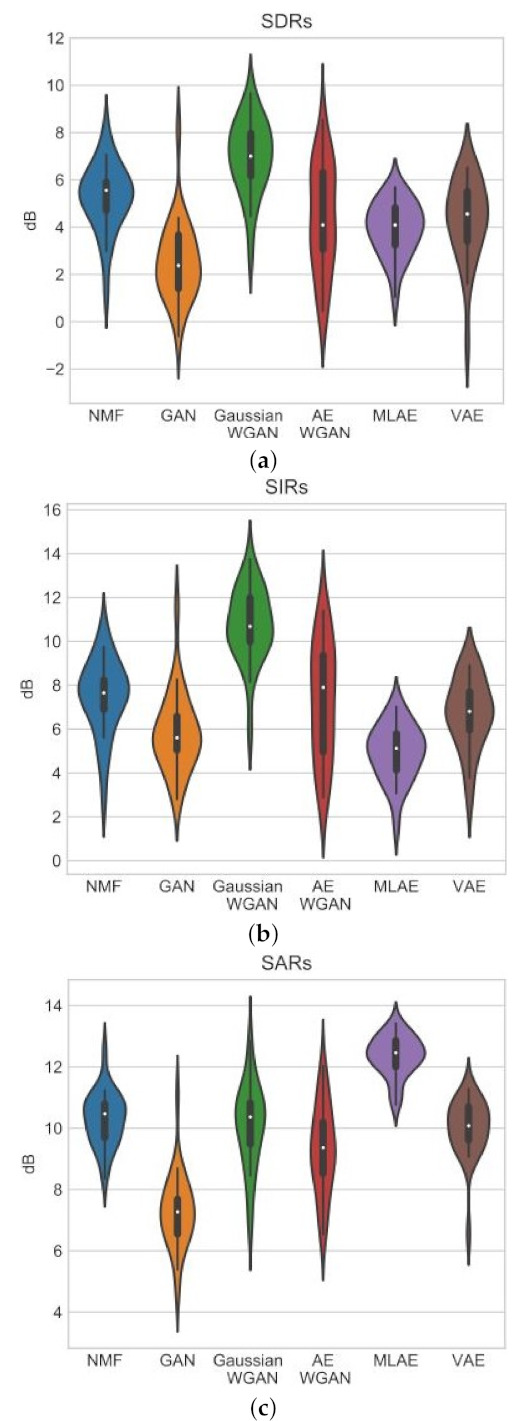
The results for the experiments from paper [114]; the graphs are taken directly from this paper. (**a**) These are the results for the SAR. (**b**) These are the results for the SDR. (**c**) These are the results for the SIR.

$S_{\mathrm{mixed}\;}$ is the magnitude spectra of the mixed signal, and phase is the phase of the mixture signal. $S_{m}$ and $S_{f}$ are the reconstructed estimate of male magnitude spectra and reconstructed estimate of female magnitude spectra. ${\hat{x}}_{k}\left(t\right)$ is the reconstructed signal. iSTFT denotes the inverse STFT. ⊙ denotes element wise multiplication. The experiments show that NNMF and the VAE methods result in similar SDR, SIR and SAR. The Gaussian WGAN has a better SIR and SDR than the NNMF and the VAE. The MLAE has a superior SAR to all the other models. Figure 17 shows the results for the experiments.

In [116], the researchers used two data sets, TIMIT and VIVOS. TIMIT is a speech corpus for American English; it has 8 dialects and 630 speakers. Each speaker speaks 10 sentences. In the experiment, they used all eight dialects. Background noise was also used, particularly trumpet sounds, traffic sounds and water sounds.

**Figure 18:**
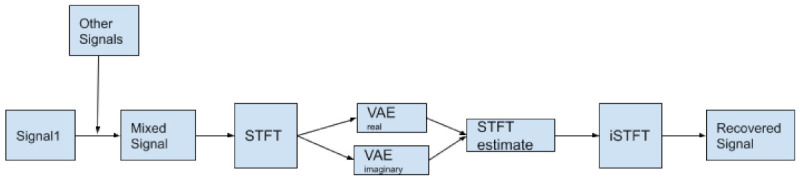
This is the architecture used by [116].

The architecture of the algorithm involved taking the STFT of the mixed signal, feeding it into a complex data based VAE, using a Chebyshev bandpass filter on the output, followed by an iSTFT to get the final reconstructed signal (Figure 18). The metrics used were SIR and SDR. There are two ways to implement a VAE for this STFT based method, to account for the complex input. One is to assume the real and imaginary part of the input are independent, there will be a VAE for the real and imaginary part respectively, and the output of the two VAEs will be rejoined again before being put into an inverse STFT (Hao Dao, Private Communications).

There were four cases: one dialect vs. one background, many dialects vs. one background, one dialect vs. many backgrounds, and many dialects vs. many backgrounds. In one dialect vs. one background, one dialect was chosen at a time, with 10 people randomly chosen, with 100 utterances total; for each dialect, the utterances were mixed with trumpet sounds and Gaussian noise. The results were shown to be good but not stable. In many dialects vs. one background, 100 utterances were chosen and mixed with trumpet sounds and Gaussian noise. The results were shown to be better than the previous case, possibly due to a different data distribution. In one dialect vs. many backgrounds, the speech data were mixed in four ways: with each background sound or all three of them. The difference in performance between the background sound was not huge, though there was a reduction when all three mixed with the speech signal. Experiments were also run with VIVOS, a speech corpus for Vietnamese language; the performance was slightly lower. They also indicate that performance depends on depth size and code size. They also compare with the ICA, regular VAE, filter banks, and wavelets, and find that their model has better SDR, SIR, and PESQ. The exact results are shown in Table 2.

In [117], source separation is achieved through class information instead of the source signal themselves using the β-VAE; this β-VAE had convolutional layers.

In [118], Convolutional Denoising Autoencoders (CDAEs) were used for monoaural source separation. Given the trained CDAEs, the magnitude spectrogram of the mixed signal is passed through all the trained CDAEs. The output of the CDAE of source *i* is the estimate ${\tilde{S}}_{i}$ of the spectrogram of source *i*. The CDAE performs better than the MLP at separating drums but is similar in separating the other components.

In [119], the multichannel conditional VAE (MCVAE) method was developed and used for semi-blind source separation with the Voice Conversion Challenge (VCC) 2018 data set. This technique has also been used with supervised determined source separation [120].

The generalized MCVAE is used to deal with multichannel audio source separation that has underdetermined conditions [121,122]. While the MVAE has good performance in source separation, the computational complexity is high, and it does not have a high source classification accuracy. The Fast MCVAE has been developed to deal with these issues [123]. The MCVAE does not perform as well under reverberant conditions, and ref. [124] works on extending the MCVAE to deal with this problem.

In [125], variational RNNs (VRNNs) were used for speech separation on the TIMIT dataset, achieving superior results over the RNN, NNMF, and DNN for SDR, SIR, and SAR.

Autoencoders and VAEs are also used for speech enhancement [126,127,128,129,130]. The goal of speech enhancement is to increase the quality of a speech signal, often by removing noise.

### 7.3. BioSignal Applications

VAEs can also be applied to biosignals, such as electrocardiogram (ECG) signals, electroencephalography (EEG) signals, and electromyography (EMG) signals.

#### 7.3.1. ECG Related Applications

**Figure 19:**
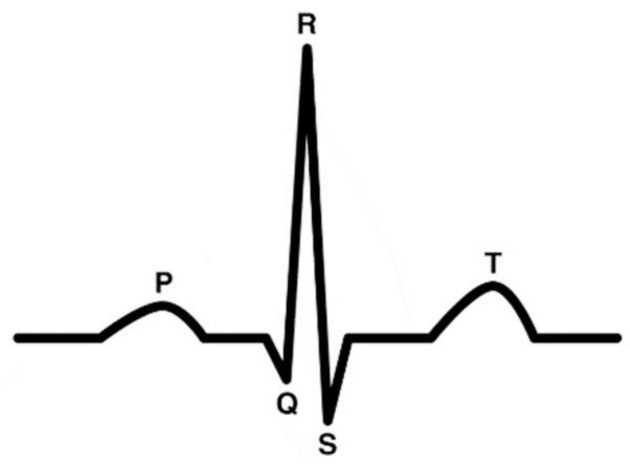
ECG waveform with P, Q, R, S, and T waves shown, taken from [133].

ECG machines measure the electrical signals from the heart; the signal recorded in an ECG machine is known as an ECG signal. The typical ECG has 12 leads; six on the arms/legs are called limb leads, and the six on the torso are called precordial leads. ECG waves can be defined as a “*positive or negative deflection from baseline that indicates a specific electrical event*” [131]. The common ECG waves are the P wave, Q wave, R wave, S wave, T wave, and U wave. A typical ECG waveform is shown in Figure 19. The frequencies of an ECG signal are in the 0–300 Hz range, though most of the information is available in the 0.5–150 Hz range [132].

Using ECGs, doctors can detect serious diseases by identifying distortions in the signal. One very important application is measuring the ECG signal of a fetus when a woman is pregnant; this is to help detect any heart problem that the fetus has. There are invasive and noninvasive methods of measuring this; due to side effects of the invasive methods, the noninvasive method is preferred. However, the mother’s ECG (MECG) signal is mixed with the baby’s ECG (FECG) signal, along with external respiratory noise. Thus, the two signals need to be separated, which is the blind source separation problem. The traditional methods to deal with fetal source separation have been ICA methods and adaptive filters such as LMS and RLS. For GANS, VAEs and AEs, it is more difficult to train the models due to the fact that we do not have a ground truth for the FECG signals. This problem can be solved by generating synthetic FECG and MECG signals from libraries such as signalz [134] and FECGSYN toolbox [135]. The average range for the beats per minute (BPM) of a pregnant woman is 80–90. The average range for the BPM of a fetus is 120–160. The MECG signal amplitude is also 2–10 times larger than the amplitude of a FECG signal. These are the important parameters needed to generate the synthetic ECG signals necessary.

**Figure 20:**
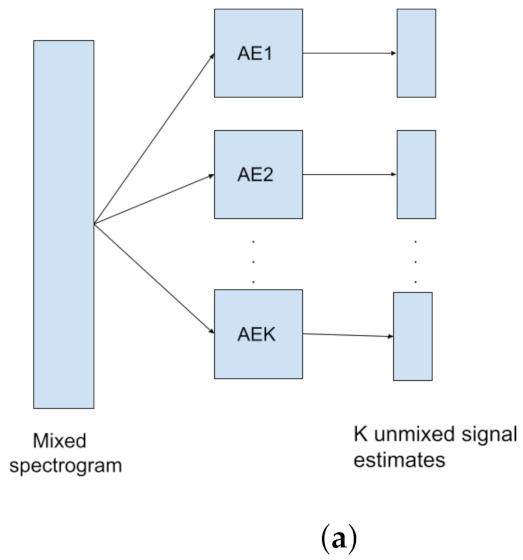

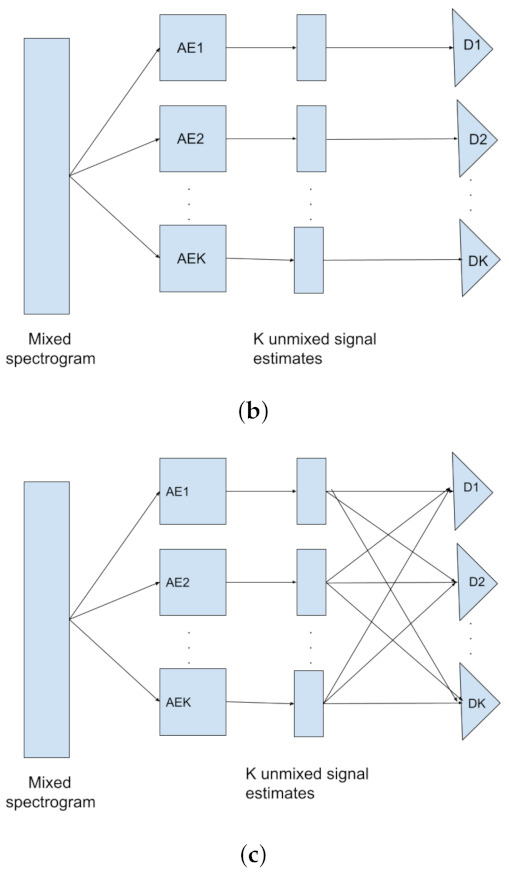
The architectures for the experiments for each model from [136]. (**a**) This is source separation using K autoencoders. (**b**) This is the CASS architecture, where each component is trained independently. (**c**) This is the CASS architecture using cross adversarial training.

While the VAE itself has not been used for fetal source separation, a similar method, the cross adversarial source separation framework (CASS), has been used. CASS is a method mixing AE and GANs for source separation tasks [136]. For each mixture component, there is an autoencoder and discriminator. The mixed signal goes into each autoencoder, whose output goes into a discriminator. Typically, each AE and GAN pair is trained independently. For each pair, the ith signal is separated, and the rest of the components in the mixture are treated as noise. Cross adversarial training is used to share information between each of those components. This was done by letting the ith discriminator to reject samples from the other components. Figure 20 shows the architectures. In their paper, the authors used two components in CASS for this particular problem. They generated synthetic FECG and MECG signals, mixed them, and added noise to simulate periodic respiratory noise. This noise consisted of random sinusoidal signals with varying frequencies and varying amplitudes. The synthetic data were converted into spectrograms before being inputted to the networks. The results are shown in Table 3. The CASS is superior to the AE model, but the CASS with cross adversarial training is inferior to CASS with training each component independently for the MECG.

Detecting distortions in ECG signals are difficult to find due to noise from disturbances, such baseline wandering, muscle shaking, and electrode movement. The VAE has been used to distinguish these ECG signals under noise conditions [137]. They used three data sets: AHA ECG database, the APNEA ECG database, and CHFDB ECG database. From these datasets, they obtained 30,000 ECG signals.

To evaluate how well the model denoised the ECG signals, noise was added to the ECG signal data. This included AWGN, salt and pepper noise, and Poisson noise (also known as shot noise). Sinusoidal signals with different amplitudes were also added to the signal to imitate baseline wandering noise. First, the ECG signal is preprocessed; this is done by using an algorithm to split the waves in segments according to the cardiac cycle. After these steps are completed, the new data are inputted into a VAE. The results showed that the VAE is as robust in the noise scenarios presented.

Morphological diagnosis, which is “*A diagnosis based on predominant lesion(s) in the tissue(s)*” [138], is one use of ECGs. Human experts typically perform better at ECG morphological recognition than deep learning methods, due to the fact that there are an insufficient amount of positive samples. In [139], a pipeline is used that involves the VQ-VAE to generate new positive samples for data augmentation purposes. A classifier was then trained using this additional synthetic data to identify ten ECG morphological abnormalities, which resulted in an increase in the F1 score for the classifier. These ten abnormalities are myocardial infarction (MI), left bundle branch block (LBBB), right bundle branch block (RBBB), left anterior fascicular block (LAFB), left atrial enlargement (LAE), right atrial enlargement (RAE), left ventricular hypertrophy (LVH), right ventricular hypertrophy (RVH), I ${}^{\circ }$ atrial ventricular block (IAVB), and pre-excitation syndrome (WPW).

Myocardial infarctions are also known as heart attacks, which can often lead to death. They need to be rapidly diagnosed to prevent deaths. Conventional methods are not very reliable and also perform poorly when applied to 6 lead ECG. In [140], a deep learning algorithm was used to detect myocardial infarction using a 6 lead ECG. They found that using a deep learning algorithm with VAE for 6-lead ECG performed better than the traditional rule-based interpretation. 425,066 ECGs from 292,152 patients were used in the study.

Deep learning can be used to classify the type of beat in an ECG signal. However, due to the black box nature of deep learning algorithms and their complexity, they are not easily adopted into clinical practice. Autoencoders have been used to reduce the complexity; the neural networks models would then use a lower dimensional embedding for the data. This solution still has problems with interpretability due to interactions between components of the embeddings. The β-VAE can be used to disentangle these interactions between components, leading to an interpretable and explainable beat embedding; this was done in [141]. They used the β-VAE to create interpretable embedding with the MIT-BIH Arrhythmia dataset. VAEs can also be used to generating an ECG signal for one cardiac cycle [142]. This is useful for data augmentation purposes. This method is relatively simple but cannot generate whole ECG signals.

In electrocardiographic imaging, recreating the heart’s electrical activity runs into numerical difficulties when using body surface potentials. A method using generative neural nets based on CVAEs have been used to tackle this problem [143].

#### 7.3.2. EEG Related Applications

EEG machines measure the electrical signals from the scalp; these are used to measure problems in the brain. A RNN based VAE has been used to improve the EEG based speech recognition systems by generating new features from raw EEG data [144]. VAEs have been used to find the latent factors for emotion recognition in multi channel EEG data [145]. These latent factors are then used as input for a sequence based model to predict emotion. Similarly, the bi-lateral variational domain adversarial neural network (BiVDANN), which uses a VAE as part of its architecture, has been used for emotion recognition from EEG signals [146]. Video game to assess cognitive abilities have been developed, with a task performance metric and EEG signals recorded; from this, a deep learning algorithm for detecting task performance from the EEG data has been developed [147]. First, this involves feature extraction, then dimensionality reduction by the VAE. The output of the VAE are used as input to an MLP, which predicts task performance.

#### 7.3.3. EMG Related Applications

An EMG machine “*measures muscle response or electrical activity in response to a nerve’s stimulation of the muscle*” [148]. They can be used to find neuromuscular problems. Upper limb prosthetics use myoelectric controllers; however, these controllers are susceptible to interface noise, which reduces performance [149]. Latent representations of muscle activation patterns have been found using supervised denoising VAE. These representations were robust to noise in single EMG channels. Latent space based deep learning classifiers have outperformed conventional LDA based classifiers.

Brain-Machine Interfaces (BMIs) can be used in paralyzed patients to help return their voluntary movement; they can do this by extracting information from neural signals. However, the interface has performance issues with this method. Using latent representations through methods like the VAE has improved the performance of the BMI [150].

## 8. Experiments

### 8.1. Experiment Setup and Data

We focused specifically on speech source separation experiments. We used the TIMIT dataset for the input [115]. Two types of speakers, male and female, were used; each of the recordings was 2–4 s. We normalized the speech signals, then combined male signals with female signals to create 90 mixed signals. Eighty signals were used for training, 10 were used for testing. We used three models: the VAE, ITL-AE, and β-VAE. For all three models, we used fully connected layers.

First, we will go over the VAE setup. There is a VAE for each speaker; thus, for a mixed signal that mixes a female and male speaker, we need two VAEs. The input to the VAE is a normalized spectrogram. The training label is the ground truth spectrogram of the original signal that we are trying to obtain. The signal is reconstructed via the Wiener filtering equation, from Equation (90). The metrics used SDR, SIR, and SAR, evaluated using the BSS Eval Toolbox [112]. This setup is repeated for the β-VAE and ITL-AE.

### 8.2. Results

#### 8.2.1. Hyperparameter Tuning

Normalizing the spectrogram was necessary for the loss function to not explode. We also found that the Gaussian distribution that generates ϵ had to have a standard deviation of 0.01. Parameters that we varied include window and overlap size for the STFT, latent variable code size, and batch size.

**Figure 21:**
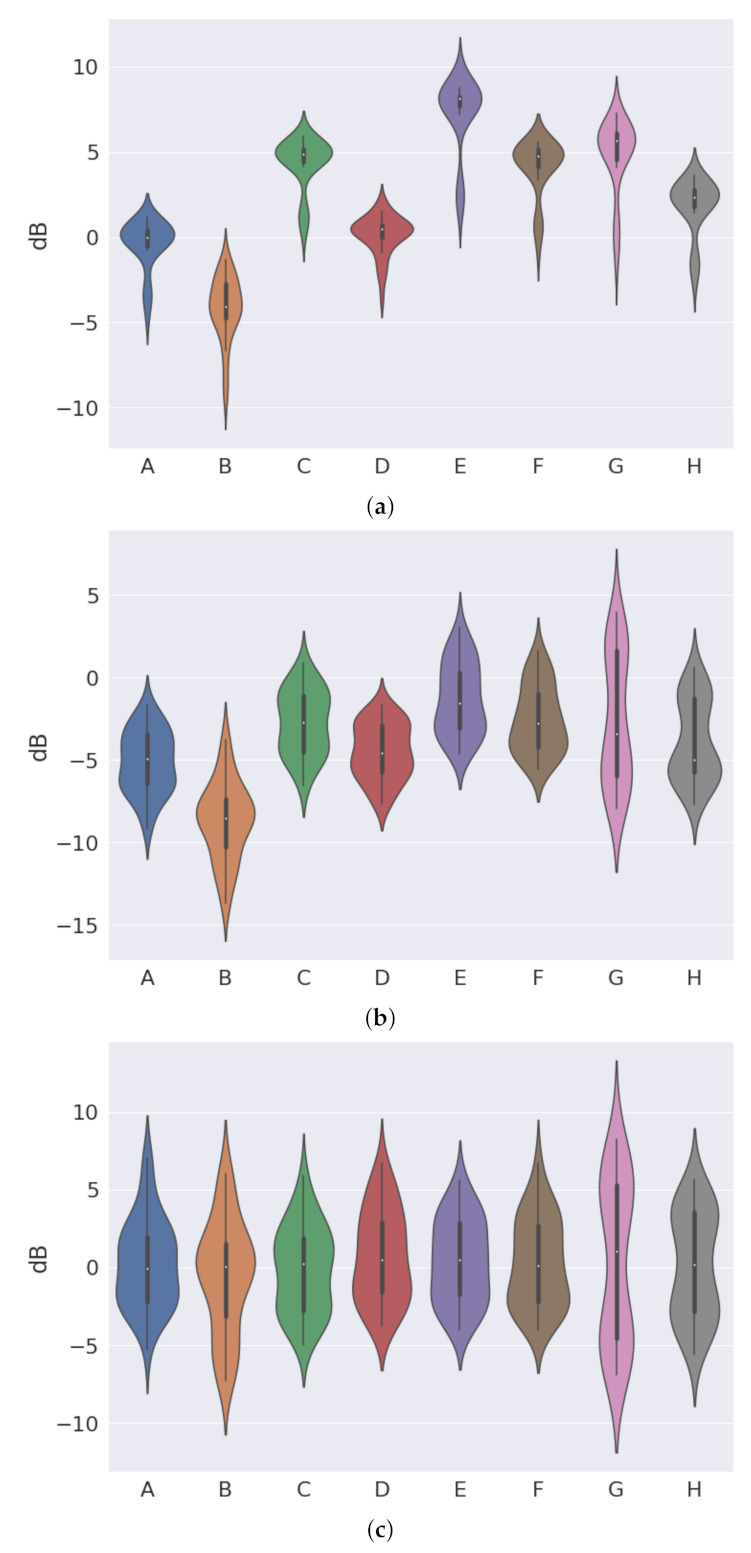
These are the results of varying the resolution and window size, in dB. For (window size, overlap size) pairs, A is (64 ms, 16 ms), B is (64 ms, 32 ms), C is (32 ms, 8 ms), D is (32 ms, 16 ms), E is (16 ms, 8 ms), F is (16 ms, 4 ms), G is (8 ms, 4 ms) and H is (8 ms, 2 ms) (**a**) These are the results for the SAR. (**b**) These are the results for the SDR. (**c**) These are the results for the SIR.

Figure 21 shows the results of varying the window size and overlap size with violin plots. The encoding layers had the value [M, 256, 256, 128], and the decoding layers had the value [256, 256, M], with M being the number of spectral frames, which depends on the choice of the STFT parameters. Using a 64 ms window size fared worse than a 32 ms window and a 16 ms window. Using 64 ms window with 32 ms overlap size resulted in a better SAR than using 64 ms window with 16 ms overlap size. Using a 16 ms window size with 8 ms overlap size had the best overall results by a wide margin. Using a 16 ms window size with 4 ms overlap size had worse results than using a 16 ms window size with 8 ms overlap size. When the window size went down to 8 ms, the results became worse than 16 ms window size.

We varied the latent code size d. The encoder layers are [129-128-d], and the decoding layers are [128-129], with the window size being 256 ms and overlap size is 128 ms. The experiment found no clear difference in SIR, SDR, and SAR. We also experiment between the batch sizes in 1, 17, 34, 70. For batch sizes 17, 34, and 70, there was no clear difference between varying the batch size. Using batch size 1 increased the time while not changing the performance.

For the β-VAE, β was a hyperparameter to be tuned. For β >1, there is no discernible difference between the various β ’s.

For the ITL-AE, the hyperparameter that we varied was the latent code size. We varied the latent code size in the range [32, 64, 128, 256]. There was no discernible difference between the various latent codes.

#### 8.2.2. Final Results

**Figure 22:**
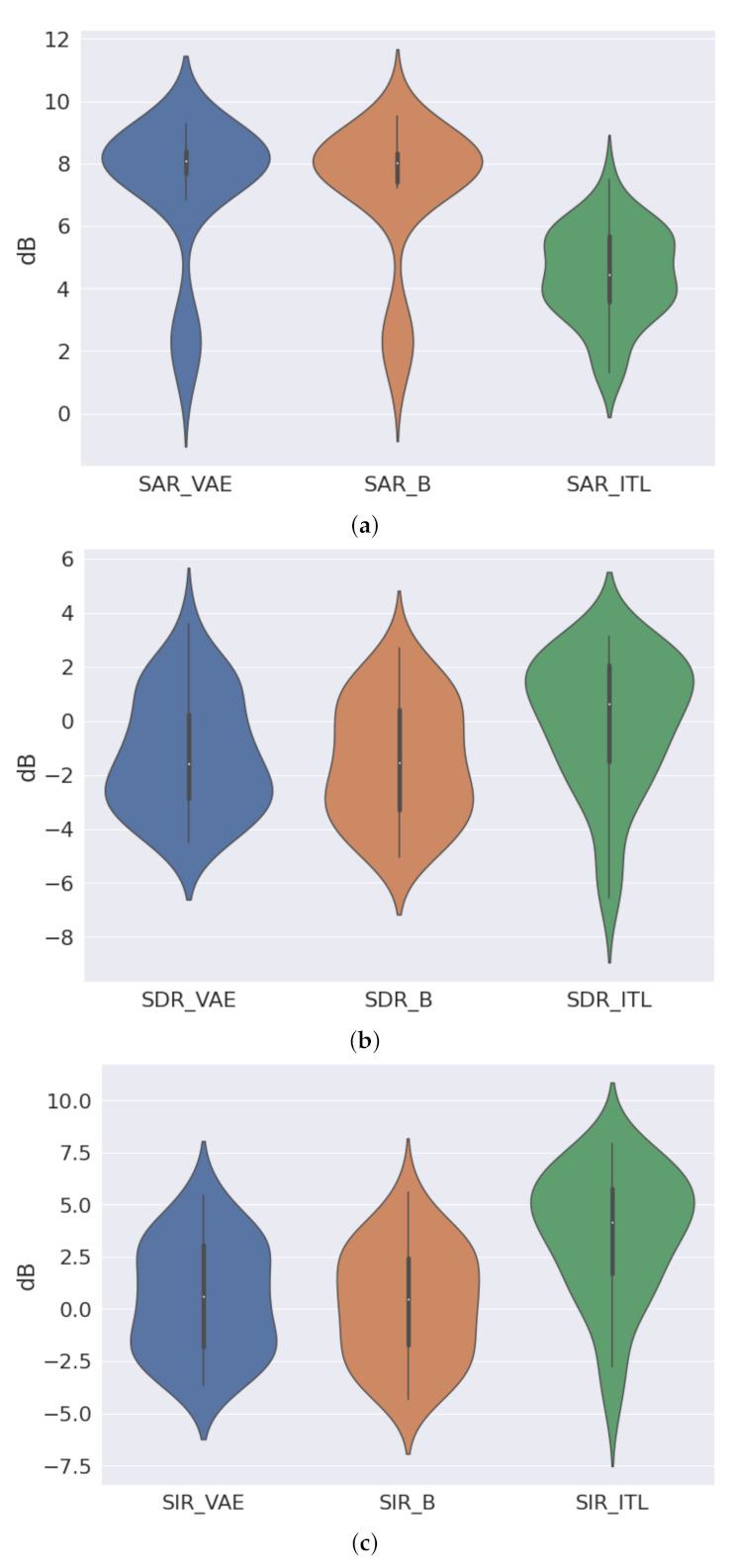
The results for the experiments for each model are shown here, in dB. (**a**) These are the results for the SAR. (**b**) These are the results for the SDR. (**c**) These are the results for the SIR.

In Figure 22, we compare the results from the three different models using violin plots. The VAE and β-VAE have similar results. The ITL-AE has a worse SAR, similar SDR, and a better SIR.

## 9. Conclusions

In this paper, first, we provided a detailed tutorial on the original variational autoencoder model. After that, we outlined the major problems of the vanilla VAE and the latest research on resolving these issues. These problems include posterior collapse, variance loss/image blurriness, disentanglement, the soap bubble effect, and the balancing issue.

Then, we comprehensively surveyed many important variations of the VAE. We can organize most of the variations into the following four groups:

(1) Regularizing Posterior Variant:We can regularize the posterior distribution to improve the disentanglement, like with the β-VAE and INFOVAE.
(2) Prior/Posterior variants: We can change the prior or posterior, like with the hyperspherical VAE, ρ-VAE, and the VQ-VAE. The hyperspherical VAE uses a vmF for the posterior and a uniform for the prior, which makes it superior for a hyperspherical latent space. The ρ-VAE uses an AR(1) Gaussian process as its posterior, which leads to superior results over a vanilla VAE in terms of the loss function.
(3) Architectural Changes: There are many potential architectural changes. The VAE-GAN and Adversarial Autoencoder take inspiration from both the VAE and GAN, and as a result mitigate the downsides of both frameworks. Combining the VAE framework with neural autoregressive models has created more flexible inference networks; the PixelVAE combines the PixelCNN with the VAE, allowing it to capture both small details and global structure. The conditional VAE uses a conditional likelihood instead of likelihood; it has been key to the MCVAE method for source separation. The MMVAE is better at dealing with data that have multiple modalities.
(4) Other Variations: Variance reduction of the VAE has been achieved through the STL estimator. Methods such as the IWAE use importance sampling to achieve a tighter lower bound for the loss function; variations of the IWAE using a DreG estimator or STL estimator have also reduced variance.

The applications of VAEs for generating images, speech/audio and text data are well known and well studied. In our article, we decided to focus on the less well known applications that VAEs can be used for, specifically in signal processing/time series analysis. In finance, we highlighted the use of the VAE in generation and completion of volatility surfaces for options, along with dimensionality reduction use cases. In the speech source separation subsection, we summarized the research on using the VAE framework for source separation in speech signals. We reviewed the use of the VAE framework for dimensionality reduction, generating disentangled interpretable features, and data generation for biosignals such as EEG, ECG, and EMG signals.

Some of the future potential areas of research for VAEs are:

(1) Disentanglement Representations: While many VAE extensions are state of the art for disentanglement, there is still the problem that they do not learn the disentangled representation in a truly unsupervised manner.
(2) Data Generation in Finance: VAEs are relatively unused in finance applications compared to other fields. For generating data, the VAE framework has been studied thoroughly and has had amazing results for natural images and audio data. Research into generating finance related data, such as volatility surfaces, is relatively unexplored, as we have only found two papers on this topic.
(3) Source Separation for Biosignals and Images: While speech source separation using the VAE framework has been heavily explored, the literature on biosignal source separation and image source separation with VAE is sparse. We see these as strong candidates for exploring the powerful capabilities of the VAE.

## Figures and Tables

**Table 1:** Results from [105] comparing the Heston Model with the VAE for six currency pairs. The units are in Mean Absolute Error (MAE), and the VAE has a latent code size of 4.

Currency Pair	Heston Model	VAE Model
AUD/USD	56.6	33.6
USD/CAD	35.3	32.5
EUR/USD	32.2	30.9
GBP/USD	47.5	34.0
USD/JPY	58.4	38.2
USD/MXN	92.2	56.7

**Table 2:** Results from [116], comparing the paper’s approach with other methods.

Group	Model	SDR (dB)	SIR (dB)	PESQ
1	Wavelet	7.56	16.22	-
	Time-Frequency filter bank	9.47	1.09	-
2	ICA	5.98	11.92	-
	VAE	9.47	-	2.37
3	VAE	9.47	-	2.35
Paper Approach	VAE	9.08	14.76	2.02
	BPF	6.87	10.01	0.97
	VAE + BPF	12.99	15.02	2.41

**Table 3:** Results from [136]. $L_{p}$ norm errors of different frameworks after training. The three frameworks were the baseline AE design, CASS (training each component independently), and CASS with cross adversarial training.

Maternal Method	$L_{1}$ Error	$L_{2}$ Error	$L_{\infty }$ Error
Baseline AE	0.45158	0.53502	0.85077
CASS	0.40672	0.47942	0.77370
CASS with Cross Training	0.40994	0.48082	0.77911
**Fetal Method**	$L_{1}$ **Error**	$L_{2}$ **Error**	$L_{\infty }$ **Error**
Baseline AE	0.49818	0.57435	0.80708
CASS	0.37387	0.46627	0.75402
CASS with Cross Training	0.37218	0.45848	0.74462

## Data Availability

The TIMIT dataset is available in a publicly accessible repository.
